# Prognostic and immunotherapeutic potential of disulfidptosis-associated signature in pancreatic cancer

**DOI:** 10.3389/fimmu.2025.1568976

**Published:** 2025-03-26

**Authors:** Ran Cui, Gaoming Wang, Renhao Hu, Yongkun Wang, Huiling Mu, Yanxiang Song, Bo Chen, Xiaohua Jiang

**Affiliations:** ^1^ Department of Hepatopancreatobiliary Surgery, Shanghai East Hospital, School of Medicine, Tongji University, Shanghai, China; ^2^ Department of Biliary-Pancreatic Surgery, Renji Hospital Affiliated to Shanghai Jiao Tong University School of Medicine, Shanghai, China; ^3^ Department of Biobank, Shanghai East Hospital, School of Medicine, Tongji University, Shanghai, China

**Keywords:** disulfidptosis, pancreatic adenocarcinoma, prognostic signature, tumor microenvironment, immunotherapy

## Abstract

Disulfidptosis is a newly discovered formation of programmed cell death. However, the significance of disulfidptosis in pancreatic adenocarcinoma remains unclear. Our investigation aims to elucidate the significance of disulfidptosis in pancreatic ductal adenocarcinoma by integrating diverse datasets, including bulk RNA sequencing data, microarray profiles, single-cell transcriptome profiles, spatial transcriptome data, and biospecimens. Utilizing various bioinformatics tools, we screened disulfidptosis-related genes based on single-cell RNA sequencing profiles, subsequently validating them through enrichment analysis. An 8-gene disulfidptosis-related prognostic signature was established by constructing massive LASSO-Cox regression models and validated by multiple external PDAC cohorts. Evaluation methods, such as Kaplan-Meier curves, ROC curves, time-dependent ROC curves, and decision curve analysis, were employed to assess the prognostic signature’s reliability. High disulfidptosis-related scores were associated with a poorer prognosis and diminished sensitivity to immune checkpoint blockade. Further investigation uncovered that the potential components of elevated DPS involve malignant tumor hallmarks, extensive interactions between myCAFs and tumor cells, and the exclusion of immune cells. Cell-cell communication analysis highlighted myCAFs’ role in signaling, potentially influencing tumor cells towards increased malignancy through collagen, laminin, and FN1 signaling networks. Spatial transcriptome analysis confirmed the crosstalk between myCAFs and tumor cells. Biospecimens including 20 pairs of PDAC samples and adjacent normal tissues further demonstrated the robustness of DPS and its correlation with CAF markers. In conclusion, our study introduces a novel disulfidptosis-related signature with high efficacy in patient risk stratification, which has the ability to predict the sensitivity to immune checkpoint blockade.

## 1 Introduction

According to the 2023 American cancer statistics, pancreatic cancer is the third leading cause of cancer death in men and women combined (1). Pancreatic ductal adenocarcinoma (PDAC) represents the predominant pathological subtype of pancreatic cancer, characterized by high aggressiveness and heterogeneity. Owing to the features of insidious onset, a significant number of patients miss the opportunity for anatomical excision upon diagnosis, contributing to the exceptionally poor prognosis of PDAC, with a 5-year survival rate hovering around 8% (1, 2). However, surgery alone is not enough for the treatment of PDAC, as more than 90% of patients suffer relapse and die without additional therapy (3). Chemotherapeutic strategies including 5-fluorouracil/leucovorin with irinotecan and oxaliplatin (FOLFIRINOX) and gemcitabine/nab-paclitaxel can help improve the prognosis of PDAC, while the development of chemoresistance and serious side effects greatly limit the clinical benefits (4). Targeted therapy, represented by small molecular inhibitors and immune checkpoint inhibitors (ICIs) represented by PD-1 inhibitors, offer potential avenues for cancer cure. However, neither has shown exciting therapeutic effects in PDAC (5). Hence, there is an urgent need to reveal novel biomarkers to help clinical decision-making and improve the survival of PDAC patients.

Cell death, a fundamental process in all living organisms, has witnessed recent discoveries of novel modes, including disulfidptosis (6), ferroptosis (7), and cuproptosis (8). These not only regulate distinct cell fates but also provide innovative ideas for overcoming the bottleneck in cancer treatment. Among them, ferroptosis is featured by the accumulation of excessive iron ions and activated lipid peroxidation resulting from the dysregulation of iron ion transport and metabolism within cells (7). Recent studies have revealed the relationships between ferroptosis and cancer cell metabolism, proliferation, and the tumor microenvironment (TME) and suggest that targeting ferroptosis has potential as a new approach for anticancer therapy (9). In contrast, disulfidptosis, a recently identified cell death type, is classified as a metabolic-related regulated cell death (10). Solute carrier family 7 member 11 (SLC7A11), belongs to a heteromeric, sodium-independent, anionic amino acid transport system that is highly specific for cysteine and glutamate and plays a central role in disulfidptosis initiation. Nicotinamide adenine dinucleotide phosphate (NADPH) serves as a critical electron donor, providing the reducing power for anabolic reactions and redox balance (11). In situations of glucose starvation, limited NADPH production from the pentose phosphate pathway occurs, leading to imminent reducing power exhaustion. At the same time, massive uptake of cystine mediated by SLC7A11 leads to the accumulation of disulfide bonds between actin cytoskeleton proteins and the collapse of the actin filament network after NADPH depletion, ultimately triggering disulfidptosis (6, 10). Hence, cells with a high tendency of disulfidptosis are characterized by high expression of SLC7A11 and unstable cellular redox state. Targeting disulfidptosis may open a new field in cancer treatment. However, difficulties like the unclear relationship between disulfidptosis and prognosis, insufficient understanding of mechanisms, and lack of drugs to selectively induce tumor cell disulfidptosis remain to be solved.

**Figure 1:**
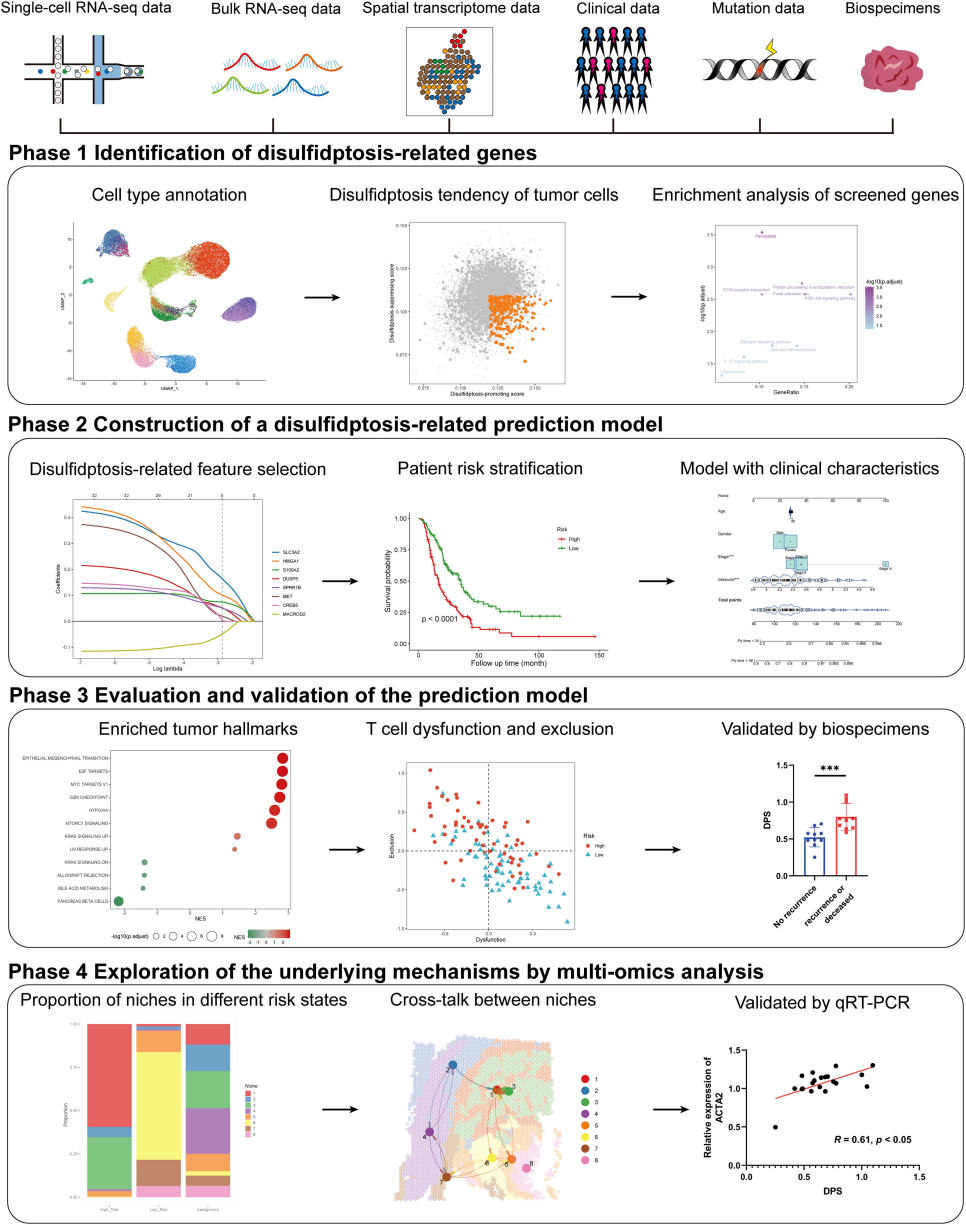
Schematic of the study. The framework of the four-phase study. DPS, disulfidptosis-related score; KRAS, kirsten rat sarcoma viral oncogene homolog.

In the present study, we aim to develop a general and robust disulfidptosis-related prognostic signature and explore its potential components in several aspects of PDAC through comprehensive multi-omics analysis and experimental validation (**Figure 1**). Our study provided a novel approach to help improve the prognostic assessment of PDAC patients, which highlights the potential of disulfidptosis in clinical application and may help clinical decision-making. Besides, we created an online tool for the easy application of our disulfidptosis-associated signature (https://mingshsmu.shinyapps.io/dps_pdac/). Additionally, our findings reveal critical communications between myofibroblastic cancer-associated fibroblasts (myCAFs) and PDAC tumor cells, correlating with poor prognosis and insensitivity to immunotherapy.

## 2 Materials and methods

### 2.1 Data collection and preparation

The detailed results of CRISPR-Cas9 screening for disulfidptosis were acquired from ref (6). A total of 1,250 PDAC samples with simultaneous transcriptome profiles and corresponding prognostic information of patients were collected from 10 distinct datasets via various databases including The Cancer Genome Atlas (TCGA), the Gene Expression Omnibus (GEO), the International Cancer Genome Consortium (ICGC) data portal, and the Omics Discovery Index (OmicsDI). Among them, 582 samples were detected with RNA-array platform (41 samples from GSE28735 (12), 63 samples from GSE57495 (13), 64 samples from GSE62452 (14), 79 samples from GSE85916, 47 samples from GSE102238 (15), and 288 samples from E-MTAB-6134 (16)), while 541 samples were profiled using RNA-sequencing technology (141 samples from TCGA-PAAD, 186 samples from PACA-CA, and 87 samples from PACA-AU (17), 127 samples from CPTAC-PDAC (18)). In detail, data from five datasets including GSE28735, GSE57495, GSE62452, GSE85916, and GSE102238 were accessed through the GEO database (https://www.ncbi.nlm.nih.gov/geo/). E-MTAB-6134 dataset was obtained from OmicsDI (https://www.omicsdi.org/). The RNA-sequencing profiles and corresponding survival information of PDAC patients in the TCGA-PAAD dataset were acquired from the University of North Carolina TCGA genome characterization center (http://xena.ucsc.edu/), while the somatic mutation data were downloaded via “TCGAmutations” R package (19). In particular, clinicopathological features were obtained from the cBioPortal website (https://www.cbioportal.org/). PACA-CA and PACA-AU datasets were accessed through the ICGC data portal (https://dcc.icgc.org/). The genetic mutation data, transcriptome data, and clinical information of the CPTAC-PDAC cohort were downloaded from LinkedOmics (https://www.linkedomics.org/data_download/CPTAC-PDAC/). The detailed criteria for inclusion of patients enrolled in this research were as follows: 1) histologically confirmed PDAC and simultaneously available information on transcriptome profiles and survival information. To reduce bias, patients with a follow-up time of less than 1 month were excluded. For bulk transcriptome data preprocessing, gene expression values detected by RNA-array were log2(x+1) transformed, while fragments per kilobase million (FPKM) generated from RNA-sequencing were converted into transcripts per million (TPM) and subsequently transformed as log2(TPM+1). Batch effects among different RNA arrays and several RNA-sequencing datasets were removed using the “ComBat” function with the parametric empirical Bayes frameworks from the “sva” R package, respectively.

The single-cell RNA-sequencing profiles of 24 PDAC tumor samples and 11 control pancreases as well as annotations of each cell were obtained from the Genome Sequence Archive (GSA) via the accession ID of CRA001160 (20). For data preparation and quality control, possible “doublets” were detected and removed via the “DoubletFinder” R package setting the doublet rate parameter as 8% (21). Quality control criteria were as follows: 1) cells had either fewer than 400 or greater than 50,000 RNA counts, 2) less than 100 or more than 8,000 RNA features, or 3) over 10% RNA features derived from the mitochondrial genome were removed. The remaining 52,534 cells were used for this study. Two PDAC samples with hematoxylin and eosin (H&E) staining and spatial transcriptome profiles were downloaded from GSE211895 (22). In total, 2,204 spots and 3,232 spots were available for further analysis, respectively.

### 2.2 Differential expression analysis

For bulk RNA-sequencing data, differentially expressed genes (DEGs) between two groups were analyzed through the “DESeq2” R package based on the raw count matrix. For normalized RNA-array values or TPM values, the “limma” R package was utilized to calculate the DEGs. Genes with the absolute value of logFC (fold change) > 1 and adjusted P-value < 0.05 were considered significant. Markers for cell clusters or spatial niches were calculated via the “FindMarkers” function from the “Seurat” R package (version 4.3.0) with default parameters (23). Particularly, only positive markers were kept for screening disulfidptosis-related genes.

### 2.3 Functional enrichment analysis

Gene Ontology (GO) items and Kyoto Encyclopedia of Genes and Genomes (KEGG) pathways were analyzed based on the DEGs through the “clusterProfiler” R package (24). In particular, the top 200 DEGs were used for enrichment analysis if the number of DEGs exceeded 200. Gene set enrichment analysis (GSEA) was conducted based on a list of DEGs and their values of logFC by setting the annotated gene sets in “h.all.v2023.1.Hs.symbols”, “c2.cp.kegg.v2023.1.Hs.symbols”, and “c5.go.v2023.1.Hs.symbols” obtained from the Molecular Signatures Database (MSigDB, https://www.gsea-msigdb.org/gsea/msigdb/) as reference. Enriched items, pathways, and hallmarks with an adjusted p-value < 0.05 were considered statistically significant.

### 2.4 Construction and validation of prognostic models and nomograms

A total of three steps were conducted to construct prognostic models. Step one, univariate Cox regression analysis was conducted based on the integrated RNA-arrays as well as the disulfidptosis-related genes. Genes with a p-value less than 0.05 were considered significant and named disulfidptosis-related prognostic genes. In step two, the integrated RNA arrays including the expression profiles of 582 PDAC samples were separated into a training set (n = 291) and an internal validation set (n = 291) at a 1:1 ratio with the assistance of the “caret” R package. In step three, the Least Absolute Shrinkage and Selection Operator (LASSO) regression algorithm with a minimum 10-fold cross-validation was conducted to build prognostic models based on the training set and disulfidptosis-related prognostic genes. Genes and their non-zero coefficients (β) were extracted from the LASSO regression model and were subsequently used to calculate the disulfidptosis-related score (DPS) of each PDAC patient using the following formula:


$$\text{DPS}=\sum_{\text{i}=1}^{\text{n}}{\text{Exp}}_{\text{gene}\_\text{i}}\;\times {\text{ β}}_{\text{gene}\_\text{i}}$$

To evaluate the prognostic significance of DPS, several distinct PDAC cohorts with either overall survival or disease-free survival were employed. Patients in each cohort were assigned into high-DPS (H-DPS) and low-DPS (L-DPS) groups according to the optimal DPS cutoff. Kaplan-Meier curves and multivariate Cox regression analysis were employed to evaluate the risk stratification performance of the DPS. Nomograms integrating with the DPS and clinicopathological features were established on the basis of multivariate Cox regression analysis. The receiver operating characteristic (ROC) curves and time-dependent area under the curve (AUC) were introduced to assess the robustness of DPS. Decision curve analysis (DCA) was utilized to estimate the survival net benefits of each variable.

### 2.5 Mutation analysis

Somatic mutation data were processed with the “maftools” R package (25). The mutational rates of genes were calculated and displayed via the “oncoplot” function. Since mutant *KRAS* is a critical dominant driver for PDAC tumorigenesis and development, the mutation types and rates of *KRAS* were extracted and compared between H-DPS and L-DPS groups.

### 2.6 Prediction of the sensitivity to immune checkpoint blockade

The Tumor Immune Dysfunction and Exclusion (TIDE) algorithm, developed through modeling two primary mechanisms of tumor immune evasion for predicting the response to immune checkpoint blockade (ICB) (26), was utilized to predict the sensitivity of PDAC patients to ICB. The fractions of 22 types of tumor-infiltrating cells were estimated using the CIBERSORT algorithm (27).

### 2.7 Single-cell RNA sequencing data and spatial transcriptome data analysis

After doublets elimination and quality control, a total of 52,534 cells were used for subsequent study. The standard preprocessing workflow was as follows: 1) data were normalized using the “LogNormalize” method with the scale factor of 10,000; 2) the top 2,000 highly variable features (HVFs) were identified and subsequently scaled with regressing out the potential influence from cell cycle and percent mitochondrial content; 3) the first round of dimensionality reduction was performed using principal component analysis (PCA) based on the expression of top 2,000 HVFs; 4) the “harmony” integration algorithm was employed to minimize the batch effect based on the results of PCA; 5) the second round of dimensionality reduction for data visualization was carried out using the Uniform Manifold Approximation and Projection (UMAP) algorithm; 6) cell clusters were identified according the top 20 harmony dimensions with various resolution from 0.01 to 1.

General markers used for cell type annotation were consistent with Peng et al. (20) and Fu et al. (28) and listed as follows: *MMP7*, *TSPAN8*, *SOX9*, *LCN2* (ductal cell), *PRSS1*, *CTRB1*, *CTRB2*, *REG1B* (acinar cell), *CHGB*, *CHGA*, *INS*, *IAPP* (endocrine cell), *RGS5*, *ACTA2*, *PDGFRB*, *ADIRF* (stellate cell), *LUM*, *DCN*, *COL1A1*, *FAP* (cancer-associated fibroblast, CAF), *CDH5*, *PLVAP*, *VWF*, *CLDN5* (endothelial cell), *CD14*, *CD163*, *CD68*, *AIF1* (macrophage), *CCR7*, *FSCN1*, *XCR1*, *CLEC9A*, *CD1C*, *FCER1A* (dendritic cell), *S100A12*, *CLEC10A* (monocyte), *CD3D*, *CD3E*, *CD4*, *CD8A* (T cell), *MS4A1*, *CD79A*, *CD79B* (B cell), *MZB1*, *SDC1* (plasma cell), *CEACAM1*, *CEACAM5*, *CEACAM6*, *KRT19* (poor prognosis). Cell subclusters were identified by extracting the expression profiles of a certain type of cells and performing the standard preprocessing workflow repeatedly until all the cells were well annotated. Subclusters of fibroblasts were annotated according to the markers identified by Ela Elyada et al. and other researchers (29, 30): *ACTA2*, *COL10A1*, *POSTN*, *MMP11*, *SDC1*, *HOPX* (myofibroblastic CAF, myCAF), *APOD*, *C7*, *PTGDS*, *EGR1*, *IL6*, *CXCL12*, *CFD*, *DPT*, *HAS1* (inflammatory CAF, iCAF), *CD74*, *HLA-DRA*, *HLA-DPA1*, *HLA-DQA1* (antigen-presenting CAF, apCAF), *MPZ*, *S100B*, *LGI4*, *PLP1* (CAFs peripheral nerve cell, CAF_PN_), both *RGS5*/*ACTA2* and *PLVAP*/*VWF* (endothelial-to-mesenchymal transition CAF, CAF_EndMt_). Immune cell subclusters were annotated using the following previously reported markers: Cytotoxic CD8+ T cells (*PRF1*, *GZMA*, *GZMK*, *NKG7* with varying expression levels of exhaustion markers *LAG3*, *PDCD1*, *CTLA4*, *TIGIT*, *HAVCR2*, *TNFRSF9*), Naïve T cells (*IL7R*), T regulatory cells (*FOXP3*, *TNFRSF4*, *IKZF2*, *IL2RA*), Follicular T cells (*CD200*, *GNG4*, *CHN1*, *IGFL2*, *ITM2A*, *CPM*, *NR3C1*), CD8+ T effector memory cells (*CD8A*, *ZNF683*), CD8+ resident memory cells (*KLRK1*, *ITGAE*), NK cells (*FGFBP2*, *FCGR3A*), M1 macrophages (*C1QA*, *C1QB*, *C1QC*, lack of the expression of *CD163* and *IL10*), M2 macrophages (*CD163*, *IL10*, *C1QA*, *C1QB*, *C1QC*). The signature scores of each cell were calculated using the UCell algorithm (31). H-DPS and L-DPS-associated cells were selected using the Scissor method (32) by integrating bulk and single-cell sequencing data.

Spatial transcriptome data were divided into several spatial niches using the “BayesSpace” algorithm (33). H-DPS and L-DPS-associated spots were selected using the “Scissor” method (32) by integrating bulk sequencing and Spatial transcriptome profiles.

### 2.8 Cell communication and spatial communication analysis

Communication networks among cell types or spatial niches were analyzed and visualized using the “CellChat” R package (34). To minimize the bias, a cell type consisting of at least 30 cells or a spatial niche consisting of at least 10 spots was considered able to communicate with others. The probabilities of the communication network were quantified using the number of interactions and interaction strength. Spatial colocation analysis of ligands and receptors was used to further confirm the communication analysis.

### 2.9 Biospecimens and quantitative real-time PCR

This research was approved by the Ethics Committee of Shanghai East Hospital, School of Medicine, Tongji University (2022-212). A total of 20 pairs of PDAC samples and adjacent normal tissues were obtained from Shanghai East Hospital Biobank. All patients had signed informed consent for donating their specimens to Shanghai East Hospital Biobank. Total RNA was extracted from tissue samples using TRIpure Total RNA Extraction Reagent (ELK Biotechnology, EP013), following reversed transcribed via EntiLink™ 1st Strand cDNA Synthesis Kit (ELK Biotechnology, EQ003) according to the manufacturer’s instructions. Quantitative real-time polymerase chain reaction (qRT-PCR) was performed using EnTurbo™ SYBR Green PCR SuperMix (ELK Biotechnology, EQ001) and QuantStudio 6 Flex (Life Technologies). The qRT-PCR results were analyzed and examined as the relative mRNA levels based on cycle threshold (CT) values using the 2^-△△CT^ method. The primer sequences used are listed in **Supplementary Table S1**.

### 2.10 Statistical analysis

Statistical analyses were performed using GraphPad Prism 8 and R software (version 4.2.2). Categorical data were compared with the Chi-Squared test. The differences in gene expression levels between the two groups were analyzed using the Wilcoxon rank-sum test. Survival curves were generated using the Kaplan-Meier method, and the difference between the two groups was compared with the Log-Rank test. Correlation analyses were conducted using the Pearson correlation test. Mean values were compared using Student’s t-test. A p-value < 0.05 was considered statistically significant.

## 3 Results

### 3.1 Identification of disulfidptosis-related genes in PDAC tumor cells

**Figure 2:**
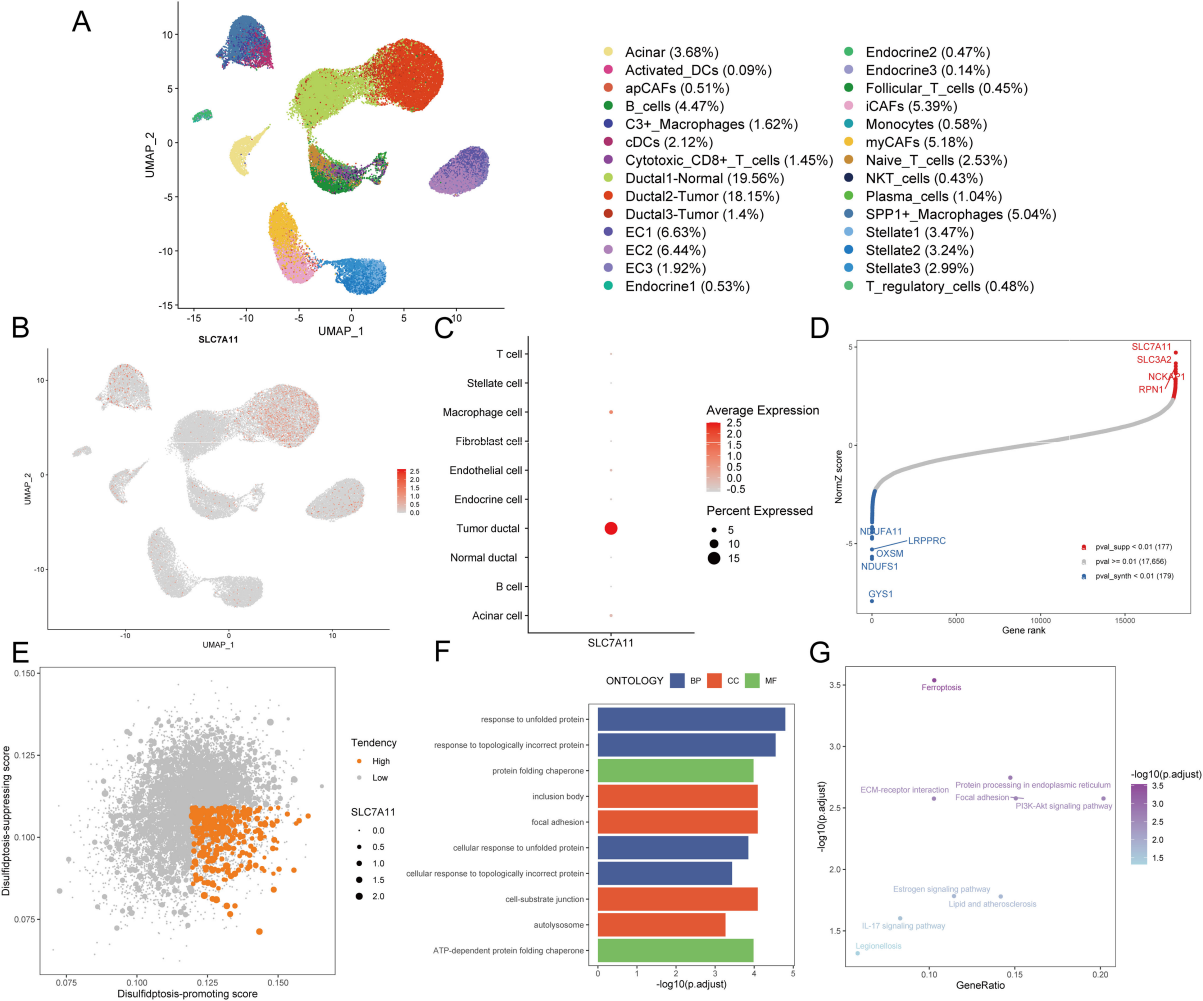
Identification of disulfidptosis-related genes. **(A)** The UMAP plot demonstrates cell subpopulations in PDAC. **(B)** The expression levels and distribution of SLC7A11 are plotted on the UMAP map. **(C)** The expression levels of SLC7A11 in each cell type are summarized by a bubble chart. **(D)** NormZ score rank plot shows the top disulfidptosis-promoting (red) and disulfidptosis-suppressing (blue) genes. CRISPR-Cas9 screening data were acquired from ref (6) **(E)** The dot plot shows the disulfidptosis tendency of each tumor cell. Tumor cells with relatively high disulfidptosis-promoting score, low disulfidptosis-suppressing score, and non-zero SCL7A11 expression were considered with high disulfidptosis tendency and represented by orange dots. Other tumor cells were considered with low disulfidptosis tendency and represented by grey dots. **(F)** The bar plot exhibits the top enriched GO terms in tumor cells with high disulfidptosis tendency. **(G)** The dot plot exhibits the enriched KEGG pathways in tumor cells with high disulfidptosis tendency. UMAP, uniform manifold approximation and projection; DC, dendritic cell; CAF, cancer-associated fibroblast; EC, endothelial cell; NKT cell, natural killer T cell; GO, gene ontology; BP, biological process; CC, cellular component; MF, molecular function; KEGG, kyoto encyclopedia of genes and genomes.

Disulfidptosis, a recently unveiled form of cell death, holds promising potential in cancer management and therapy. To screen the disulfidptosis-related genes, we obtained the single-cell RNA-sequencing data of PDAC samples and control pancreases. A total of 52,534 cells with high-quality and preliminary cell-type annotations were extracted and used for subsequent analysis. To further annotate cell subpopulations, we extracted the profiles of each cell type, found cell subclusters with high resolutions, checked cell markers, and excluded other cell types repeatedly until all cells were well annotated. As a result, a total of 28 cell subpopulations including acinar cells, normal ductal cells, two types of tumor cells, three kinds of CAFs, endothelial cells, endocrine cells, stellate cells, and various immune cell subpopulations (**Figure 2A**, **Supplementary Figures S1A-E**).

Given the crucial role of SLC7A11 in disulfidptosis, an examination of its expression across diverse cell types became imperative. Notably, only a subset of PDAC tumor cells exhibited relatively high SLC7A11 expression (**Figures 2B, C**), indicating that disulfidptosis is more likely to occur in tumor cells rather than other cell types including stromal cells and immune cells in the TME of PDAC. To assess the tendency of disulfidptosis of every tumor cell, we obtained 177 disulfidptosis-promoting genes including *SLC7A11*, *SLC3A2*, *NCKAP1*, *RPN1* et al. and 179 disulfidptosis-suppressing genes such as *GYS1*, *NDUFS1*, and *OXSM* by reanalyzing the CRISPR/Cas9 screening profiles for disulfidptosis with setting the threshold of the p-value as 0.01 (**Figure 2D**). Subsequently, disulfidptosis-associated scores of each PDAC tumor cell were calculated. Since SLC7A11 is indispensable in the process of disulfidptosis (6), we evaluated the tendency of disulfidptosis of cells using the following indexes: 1) SLC7A11 expression level, 2) disulfidptosis-promoting score, and 3) disulfidptosis-suppressing score. Consequently, 3.29% (350/10,643) of tumor cells with a relatively high disulfidptosis-promoting score, low disulfidptosis-suppressing score, and SLC7A11 expression were identified as more likely to undergo or be experiencing disulfidptosis (**Figure 2E**).

We compared the expression profiles between the aforementioned 350 tumor cells and other tumor cells, finding that 85 genes were highly expressed (average log2 fold change > 0.25 and p-value < 0.05, **Supplementary Table S2**). GO enrichment analysis demonstrated that these 85 genes were mainly enriched in terms including response to unfolded protein, response to topologically incorrect protein, and ATP-dependent protein folding chaperone, aligning with the characteristics of disulfide bond accumulation between actin cytoskeleton proteins during disulfidptosis (**Figure 2F**, **Supplementary Table S3**). The top enriched KEGG pathways such as ferroptosis and protein processing in the endoplasmic reticulum further confirmed the disorders of the cellular redox system and accumulation of abnormal proteins (**Figure 2G**, **Supplementary Table S4**). Hence, these 85 genes screened from PDAC tumor cells with high disulfidptosis tendencies were deemed reliable disulfidptosis-related genes (DPGs).

### 3.2 Construction and validation of disulfidptosis-related prognostic signatures

**Figure 3:**
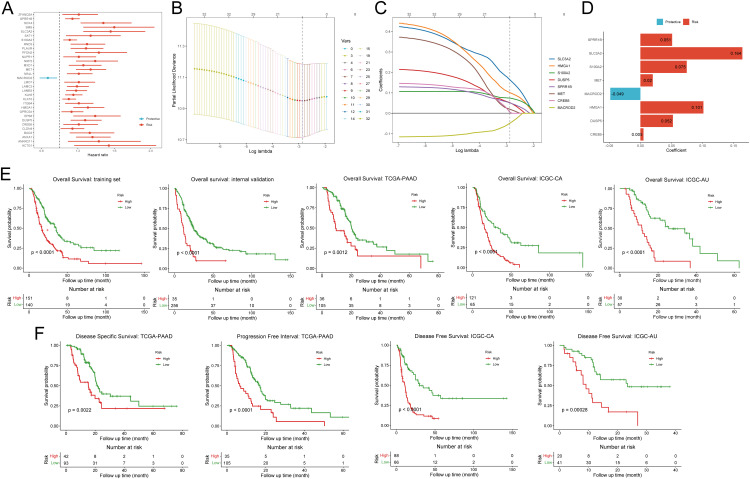
Construction and validation of the disulfidptosis-related prognostic signature. **(A)** The forest plot demonstrates the results of univariate Cox regression analysis of disulfidptosis-related genes. **(B)** The deviance varies with lambda in the Least Absolute Shrinkage and Selection Operator (LASSO)-Cox regression analysis. **(C)** The coefficient of each gene varies with lambda in LASSO-Cox regression analysis. **(D)** The bar plot shows the coefficients of eight selected genes. **(E)** Kaplan-Meier curves exhibit the prognostic value of disulfidptosis-related score on overall survival in five different cohorts. **(F)** Kaplan-Meier curves show the prognostic value of disulfidptosis-related score on disease-specific survival, progression-free survival, and disease-free survival.

For the purpose of developing clinical applications of disulfidptosis, we decided to construct a relatively general prognostic gene signature for risk stratification and survival improvement. To boost the performance of modeling, we conducted univariate Cox based on integrated bulk RNA arrays and overall survival (OS). Consequently, 32 out of 85 disulfidptosis-related genes (DPGs), comprising 31 risk genes and 1 protective gene, exhibited significant correlations with OS (p-value < 0.05, **Figure 3A**, **Supplementary Table S5**). Subsequently, we employed the LASSO Cox regression algorithm to build prognostic models based on the training set and the aforementioned 32 prognostic DPGs. After 1,000,000 attempts, we selected 801,752 as the stochastic seed and 0.05680307 as the lambda to achieve a relatively simple yet accurate predictive model (**Figure 3B**). Eventually, a panel of 8 genes including *SLC3A2*, *HMGA1*, *S100A2*, *DUSP5*, *SPRR1B*, *MET*, *CREB5*, and *MACROD2* with their non-zero coefficients were reserved for model construction and score calculation (**Figures 3C, D**; **Supplementary Table S6**). The formula for calculating the disulfidptosis-related score (DPS) for each PDAC patient was as follows: DPS = (0.16389) * Exp_SLC3A2_ + (0.10129) * Exp_HMGA1_ + (0.07452) * Exp_S100A2_ + (0.05227) * Exp_DUSP5_ + (0.05092) * Exp_SPRR1B_ + (0.01983) * Exp_MET_ + (0.00492) * Exp_CREB5_ + (-0.04851) * Exp_MACROD2_. To validate the risk stratification capability of the DPS on OS, we introduced three external PDAC cohorts including TCGA-PAAD, ICGC-CA, and ICGC-AU. As expected, the DPS successfully discriminated patients with favorable and poor survival (all p-values < 0.05, **Figure 3E**). It is worth noting that the DPS also performed well on disease-specific survival, progression-free survival, and disease-free survival (all p-values < 0.05, **Figure 3F**). Hence, the DPS we developed demonstrated robust risk stratification power and may significantly contribute to PDAC clinical management.

### 3.3 The DPS showed promoting performance in external validation cohorts

**Figure 4:**
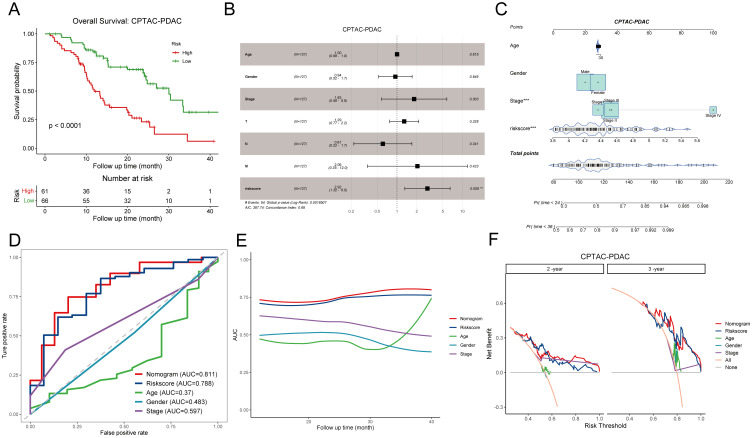
Evaluation of the prognostic significance of disulfidptosis-related score in the CPTAC-PDAC cohort. **(A)** Kaplan-Meier curves demonstrate the excellent prognostic value on overall survival in the CPTAC-PDAC cohort. **(B)** The forest plot shows a multivariate Cox regression analysis of DPS and clinical characteristics. **(C)** A nomogram for patient stratification. **(D)** ROC curves demonstrate the predictive efficiency of DPS, nomogram, and clinicopathological features at 3-year survival. **(E)** Time-dependent AUC curves of DPS, nomogram, and clinicopathological features. **(F)** DCA curves show the net benefit of DPS, nomogram, and clinicopathological features for patients in the CPTAC-PDAC cohort at 2-year survival and 3-year survival. DPS, disulfidptosis-related score; ROC curve, receiver operating characteristic curve; AUC, area under the curve; DCA, decision curve analysis. **P < 0.01; ***P < 0.001.

To rigorously assess the predictive efficiency of the disulfidptosis-related prognostic signature, we employed an additional PDAC cohort comprising 127 patients from the CPTAC. Patients were categorized into high DPS and low DPS groups according to the optimal cutoff value. Kaplan-Meier curves illustrated that patients in the low DPS group exhibited longer survival than those in the high DPS group (p-value < 0.0001, **Figure 4A**), underscoring the robust performance of the DPS. Multivariate Cox regression analysis of the DPS and clinicopathological characteristics based on the CPTAC-PDAC cohort revealed that the DPS stood out as the sole independent risk factor for the OS (p-value = 0.008, **Figure 4B**). To enhance the practical application of the DPS in clinical settings, we devised a nomogram by integrating the DPS and clinical features (**Figure 4C**). Given the notoriously poor prognosis of PDAC, we constructed ROC curves and computed the AUC values for OS with a 36-month observation time (**Figure 4D**). The AUC values of the nomogram, DPS, age, gender, stage for 3-year-survival were 0.811, 0.788, 0.37, 0.483, and 0.598, respectively, indicating that the DPS and nomogram exhibited favorable predictive capacities for survival and could serve as prognostic markers for PDAC patients. To fully understand the predictive efficiency of the DPS and nomogram, we calculated the time-dependent AUC values and fit them into smooth lines. As depicted in **Figure 4E**, the AUC values of the DPS and nomogram exceeded 0.75 at most time points, while the AUC values of age, gender, and stage were far from satisfactory. In addition, the DCA curves graphically illustrated that the net benefits at 2-year and 3-year from the nomogram and DPS were much more than those from other clinical features (**Figure 4F**). Furthermore, we conducted the same analyses on the TCGA-PAAD cohort, and the results concurred with those from the CPTAC-PDAC cohort (**Supplementary Figures S2A-D**). Taken together, the DPS and nomogram we established showed compelling performance in risk stratification and prognosis prediction, demonstrating potential for clinical application and aiding decision-making.

### 3.4 High DPS implied malignant hallmarks and immune desert

As the disulfidptosis-related prognostic signature exhibited significant prognostic relevance in PDAC, our interest turned to understand the potential components. Genetic mutations are a primary driver of tumorigenesis and development (35). We meticulously assessed and compared the top 10 altered genes between high DPS (H-DPS) and low DPS (L-DPS) groups, finding that there was little difference between these two groups not only in the mutation frequency but also in the mutant classifications (**Supplementary Figure S3A**). *KRAS* is a major oncogene in PDAC and has been found altered in more than 90% of PDAC patients (18, 36). We analyzed and compared the mutant rate of hot *KRAS* missense mutations between H-DPS and L-DPS groups. As a result, G12D, G12V, G12R, and Q61H emerged as the top 4 mutation types of *KRAS* and showed similar alteration frequency between the two groups (**Supplementary Figure S3B**). Consequently, we deduced that genetic mutations played a negligible role in contributing to DPS.

**Figure 5:**
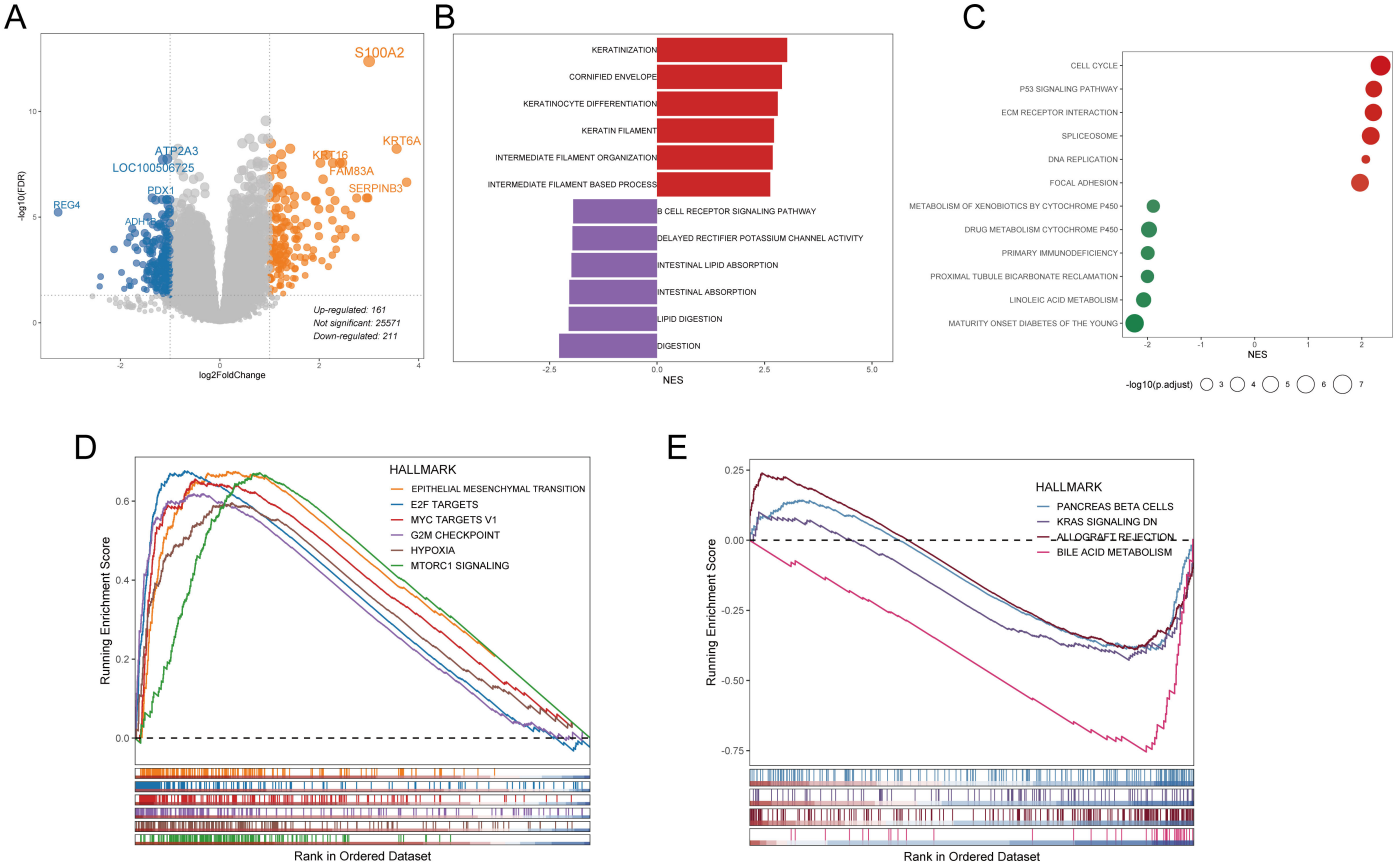
Exploration of the potential components of DPS at the transcriptome level. **(A)** Differentially expressed genes between H-DPS and L-DPS groups. **(B, C)** GSEA results exhibit the enriched GO terms **(B)** and KEGG pathways **(C)** in H-DPS and L-DPS groups. **(D, E)** GSEA curves demonstrate the enriched cancer hallmarks in the H-DPS group **(D)** and L-DPS group **(E)**. GSEA, gene set enrichment analysis.

As there was little dissimilarity in somatic mutations between H-DPS and L-DPS groups, we wondered if there existed a discrepancy in transcriptome and biological processes. DEG analysis revealed 161 up-regulated genes, including *S100A2*, *KRT6A*, *KRT16*, *FAM83A*, and *SERPINB3*, along with 211 down-regulated genes, including *ATP2A3*, *PDX1*, *REG4*, and *ADH1B*, in the H-DPS group compared to the L-DPS group (**Figure 5A**, **Supplementary Table S7**). Enrichment analysis based on these DEGs implied that GO terms including “keratinization”, “keratin filament”, “intermediate filament organization”, and “intermediate filament-based process”, which indicate the assembly of actin filament bundles and are closely related to disulfidptosis, were prominently characterized in the H-DPS group (**Figure 5B**). Meanwhile, GO terms like “digestion”, “intestinal absorption”, and “B cell receptor signaling pathway”, representing normal physiological processes, were prevalent in the L-DPS groups (**Figure 5B**). KEGG pathway analysis demonstrated that cell proliferation-associated signaling pathways including “cell cycle”, “ECM receptor interaction”, and “focal adhesion” were significantly enriched in the H-DPS groups, while metabolism-related pathways such as “linoleic acid metabolism”, “drug metabolism cytochrome P450”, and “metabolism of xenobiotics by cytochrome P450” were predominant in the L-DPS groups (**Figure 5C**). GSEA based on tumor hallmarks revealed that malignant hallmarks including “epithelial-mesenchymal transition”, “E2F targets”, “MYC targets V1”, and “hypoxia” were distinctly enriched in the H-DPS groups (**Figure 5D**). However, physiological biological processes such as “pancreas beta cells” and “bile acid metabolism”, as well as genes down-regulated by KRAS activation were notably found in the L-DPS group (**Figure 5E**). Taken together, our findings demonstrated that PDAC samples in the H-DPS group were charactered closely related to disulfidptosis and exhibited pronounced cancer hallmarks related to cell proliferation and metastasis.

**Figure 6:**
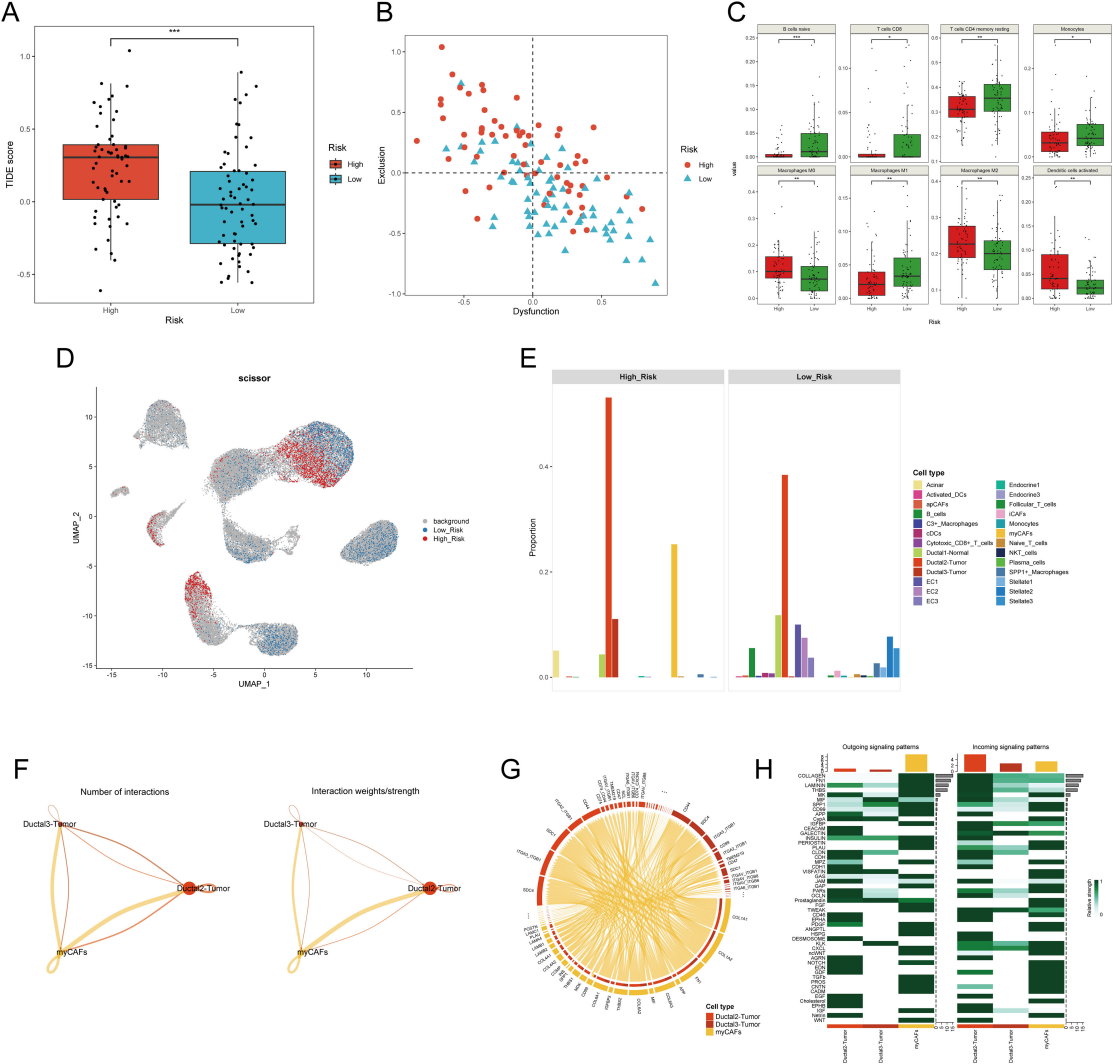
Investigation of the difference in tumor microenvironment between H-DPS and L-DPS groups. **(A)** The boxplot shows the TIDE scores of PDAC samples in H-DPS and L-DPS groups. **(B)** The point plot demonstrates the degrees of T cell dysfunction and exclusion of each PDAC sample in H-DPS and L-DPS groups. **(C)** Box plots exhibit cell types with distinct tumor-infiltrating levels between H-DPS and L-DPS groups. **(D)** The UMAP plot shows cell subpopulations associated with H-DPS and L-DPS. Cell population selection was conducted using the scissor algorithm. **(E)** Bar plots demonstrate the proportions of cell types associated with different DPS groups. **(F)** Interaction number and strength among PDAC tumor cells and myCAFs. **(G)** The chord diagram shows the top ligand-receptor pairs among PDAC tumor cells and myCAFs. **(H)** Heatmaps exhibit the relative strength of outgoing and incoming signaling patterns among PDAC tumor cells and myCAFs. TIDE, tumor immune dysfunction and exclusion; myCAF, myofibroblastic cancer-associated fibroblast.

Given that tumors are typically situated in the TME, a complex milieu comprising the extracellular matrix and diverse cell types such as fibroblasts, immune cells, and inflammatory cells (37), we desired to explore if there exists a significant difference between PDAC samples in the H-DPS and L-DPS groups since they exhibited distinct cancer hallmarks. To estimate the levels of T cell infiltration and the sensitivity to ICB, we employed the TIDE algorithm to calculate the TIDE scores which could reflect the tumor immune microenvironment. The results indicated that PDAC samples in the H-DPS group showed significantly higher levels of TIDE scores than those in the L-DPS group (**Figure 6A**; **Supplementary Table S8**), suggesting that patients in the L-DPS group might benefit more from ICB therapy, whereas patients in the H-DPS group might not be suitable for taking immune checkpoint inhibitors. Further analysis demonstrated that most PDAC samples in the H-DPS group exhibited T cell exclusion while samples in the L-DPS group showed T cell dysfunction (**Figure 6B**; **Supplementary Table S8**), suggesting lower T cell infiltration levels in the H-DPS group, contributing to reduced sensitivity to ICB. Then, we utilized the CIBERSORT algorithm to evaluate the infiltrating degrees of 22 kinds of leukocytes. Agreeing with our previous results, immune cells such as naïve B cells, CD8+ T cells, CD4+ memory resting T cells, monocytes, and M1 macrophages were significantly lower in the H-DPS groups compared to the L-DPS group (**Figure 6C**; **Supplementary Table S9**). In addition, M2 macrophages, a primary population of myeloid-derived suppressor cells (MDSCs) known for their tumor-promoting role in the TME (38), exhibited higher fractions in the H-DPS group (**Figure 6C**; **Supplementary Table S9**). These findings indicated that PDAC samples in the H-DPS group exhibited characteristics of “cold” tumors including epithelial-mesenchymal transition hallmark, elevated numbers of MDSCs, and reduced numbers of effector immune cells (39), leading to an immune desert and eventually insensitivity to ICB treatment.

### 3.5 myCAFs facilitated tumor development in high DPS samples

To gain deeper insights into the potential factors of the disulfidptosis-related prognostic signature, we utilized single-cell RNA sequencing profiles. UMAP plots showed that *S100A2*, *SPRR1B*, *MET*, and *MACROD2* were mainly expressed in PDAC tumor cells (**Supplementary Figure S4A**). Meanwhile, the expression of other model genes including *SLC3A2*, *HMGA1*, *DUSP5*, and *CREB5* exhibited distribution across various cell types (**Supplementary Figure S4A**). These findings gave few clues for further exploration. Therefore, we conducted an integrated analysis of the bulk RNA sequencing data and single-cell RNA sequencing data to investigate the potential components in higher dimensions. Utilizing the scissor algorithm with a binomial model, we identified 1,985 H-DPS-associated cells and 2,804 L-DPS-related cells (**Figure 6D**). Expression analysis demonstrated higher expression of the eight genes constituting the disulfidptosis-related prognostic model in the H-DPS-associated cells compared to the L-DPS-related cells (**Supplementary Figure S4B**), affirming the accuracy of the screened DPS-related cells. Cell proportion analysis revealed that H-DPS-related cells primarily consisted of PDAC tumor cells and myofibroblastic cancer-associated fibroblasts, with a few acinar cells and normal ductal cells (**Figure 6E**). Conversely, L-DPS-associated cells encompassed various cell types, including B cells, normal ductal cells, PDAC tumor cells, endothelial cells, inflammatory CAFs (iCAFs), stellate cells, C3+ macrophages, SPP1+ macrophages, conventional dendritic cells (cDCs), and cytotoxic CD8+ T cells (**Figure 6E**). These findings demonstrated that the TME of PDAC samples in the L-DPS group was characterized by a variety of immune and stromal cell types, which formed the basis of response to ICB treatment. However, the TME of PDAC samples in the H-DPS group lacked immune cells but comprised abundant myCAFs and tumor cells, hindering the potential for ICB therapy, and aligning with TIDE estimation. Given the intimate relationship between PDAC tissues with high DPS and myCAFs, we aimed to understand the crosstalk between them. Cellchat analysis revealed higher numbers of interactions and interaction strength from myCAFs to tumor cells compared to the reverse direction (**Figure 6F**). Detailed ligand-receptor pairs revealed that ligands like COL1A1, COL1A2, FN1, and COL6A3 from myCAFs and receptors such as SDC1, SDC4, ITGA3_ITGB1, ITGA2_ITGB1, and CD44 were the most critical signal senders and receivers in promoting tumor development (**Figure 6G**; **Supplementary Table S10**). Signaling patterns between myCAFs and cancer cells showed that myCAFs were the predominant signal senders and ductal2 tumor cells manifested as a primary signal receiver (**Figure 6H**). Pathways like Collagen, FN1, Laminin, THBS, and MK were found as key interaction patterns between myCAFs and tumor cells (**Figure 6H**). Therefore, our results suggested that myCAFs may influence the malignant transformation of tumor cells in the TME with high DPS-related features.

### 3.6 Spatial transcriptome data confirmed the communication and colocalization of myCAFs and tumor cells

**Figure 7:**
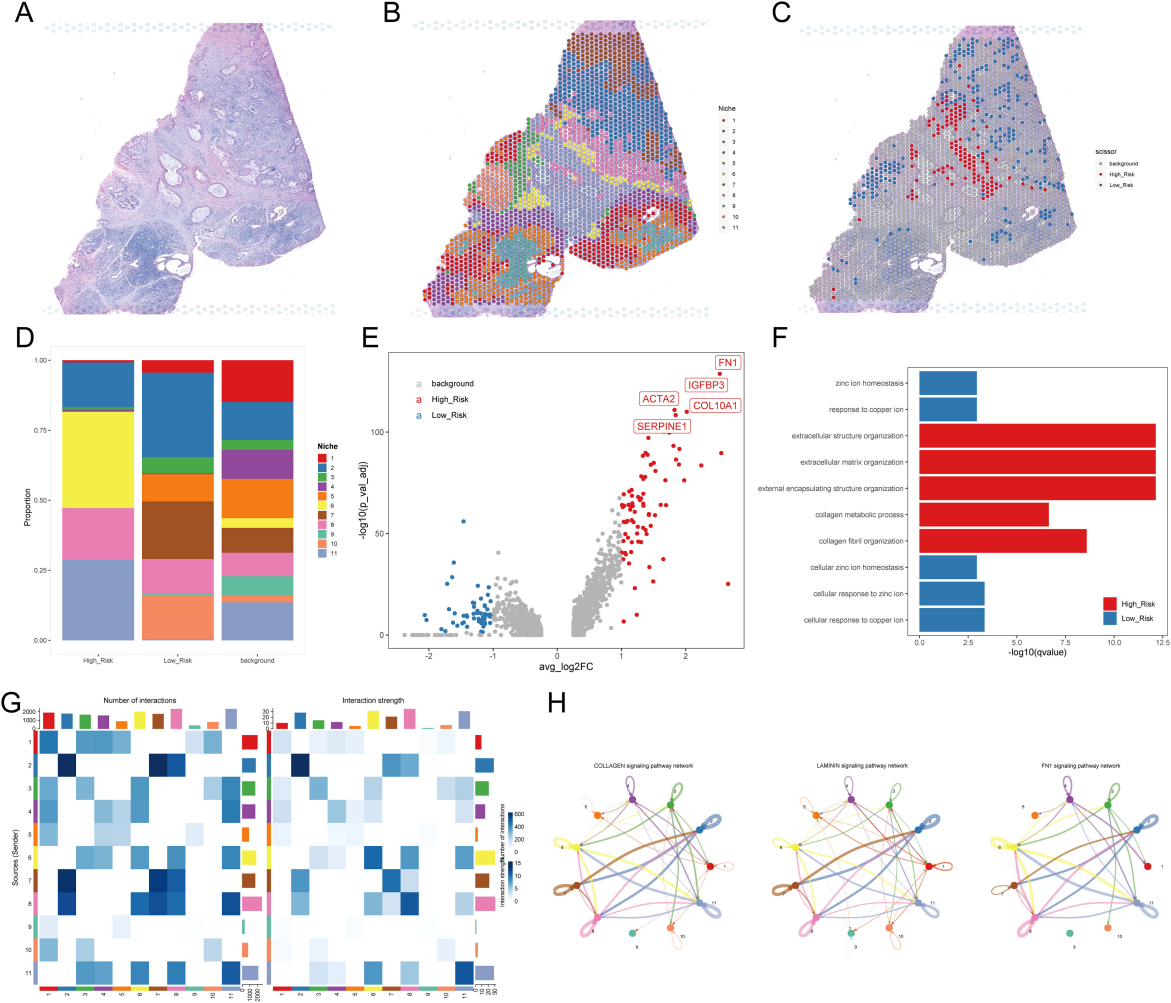
Investigation of the TME using spatial transcriptome data. **(A)** H&E staining for PDAC sample **(A, B)** Niches of PDAC sample A mapping with H&E staining. **(C)** H-DPS and L-DPS associated spots mapping with H&E staining. **(D)** Stacked bar plots exhibit the proportions of niches associated with H-DPS and L-DPS. **(E)** The volcano map shows DEGs between H-DPS and L-DPS-associated niches. The top five DEGs in H-DPS-associated niches were labeled with symbols. **(F)** Bar plots exhibit the top enriched terms in H-DPS and L-DPS-associated niches. **(G)** Heatmaps demonstrate the interaction number and strength among various niches. **(H)** The top three signaling pathway networks among niches in PDAC sample **(A)** H&E, hematoxylin and eosin.

To further validate the pivotal role of myCAFs in facilitating tumor cell development through communication with cancer cells, we obtained two PDAC samples with simultaneous spatial transcriptome profiles and H&E staining slices. In the case of sample A (**Figure 7A**), we clustered the spots into 11 niches using the BayesSpace algorithm for a more precise investigation (**Figure 7B**). The distribution of these niches aligned well with the morphology, confirming the sample’s usability and the effectiveness of the classification method. Using the scissor algorithm, we identified 125 of 1,831 spots as H-DPS-associated and 248 of 1,831 spots as L-DPS-related (**Figure 7C**). Proportion analysis demonstrated that niche 6 and niche 11 were the distinct niches associated with high DPS, while niche 5, niche 7, and niche 10 were unique to the low DPS-related spatial zones (**Figure 7D**). DEGs analysis showed that there were 82 upregulated genes like FN1, IGFBP3, COL10A1, ACTA2, and SERPINE1 and 53 downregulated genes in the high DPS-related niches compared to the low DPS-related niches (**Figure 7E**, **Supplementary Table S11**). It is worth noting that FN1 and COL10A1 are significant ligands in the FN1 and collagen signaling pathways, respectively. Meanwhile, *ACTA2*, which encodes αSMA, is an acknowledged marker of myCAFs. These results indicated that the H-DPS-related niches were highly enriched by myCAFs and extracellular matrix. Enrichment analysis demonstrated that biological processes including extracellular structure organization, extracellular matrix organization, external encapsulating structure organization, and collagen fibril organization were highlighted in the H-DPS-related niches (**Figure 7F**). Meanwhile, metal homeostases such as zinc ion homeostasis, response to copper ion, cellular zinc ion homeostasis, and cellular response to copper ion were characterized in the L-DPS-associated niches (**Figure 7F**). These results suggested that collagen, laminin, and FN1 signaling patterns constituted the predominant signaling networks. Inter-niche and intra-niche communications revealed that the interaction numbers and strength between niche 6 and niche 11 were considerable, however, the crosstalk among niche 5, niche 7, and niche 10 was minimal (**Figure 7G**). Further investigation showed that collagen, laminin, and FN1 signaling pathways were the top communication networks (**Figure 7H**), consistent with our previous results. To further confirm the crosstalk among niches, we generated the spatial expression maps of representative ligand-receptor pairs from collagen, laminin, and FN1 signaling networks (**Supplementary Figures S5A, B**). The spatial distribution of the representative ligands such as COL1A1, COL1A2, COL6A3, FN1, LAMB3, and LAMC2 exhibited consistency with the representative receptors including SDC1, SDC4, ITGA3, ITGA2, ITGAV, and ITGB1, particularly in H-DPS-related niches (**Supplementary Figures S5A, B**). Additionally, spatial locations of markers for myCAFs, iCAFs, PDAC tumors, and normal ductal cells were investigated, revealing colocalization of myCAFs and tumor cells, especially in the H-DPS-related niches and nearby zones (**Supplementary Figure S5C**). Conversely, the iCAF markers did not show any obvious consistency with cancer cell markers, but exhibited some colocalizations with the normal ductal cell markers (**Supplementary Figure S5C**). Similar analyses on sample B yielded comparable results (**Supplementary Figures S6**, **S7**, **Supplementary Table S12**). Dissection of signaling networks dissection indicated that the H-DPS-related niches (niche 6 and niche 11 in sample A, niche 1 and niche 3 in sample B) were found to play important roles as sender, receiver, mediator, and influencer in collagen, laminin, and FN1 signaling pathways (**Supplementary Figure S8**). Taken together, our findings demonstrated that the communications between myCAFs and PDAC tumors, especially in H-DPS-related niches, through signaling networks like collagen, laminin, and FN1 patterns, promoted tumor progression and reduced sensitivity to ICB treatment in patients with high DPS.

### 3.7 Validation of the disulfidptosis-related prognostic signature using biospecimens

**Figure 8:**
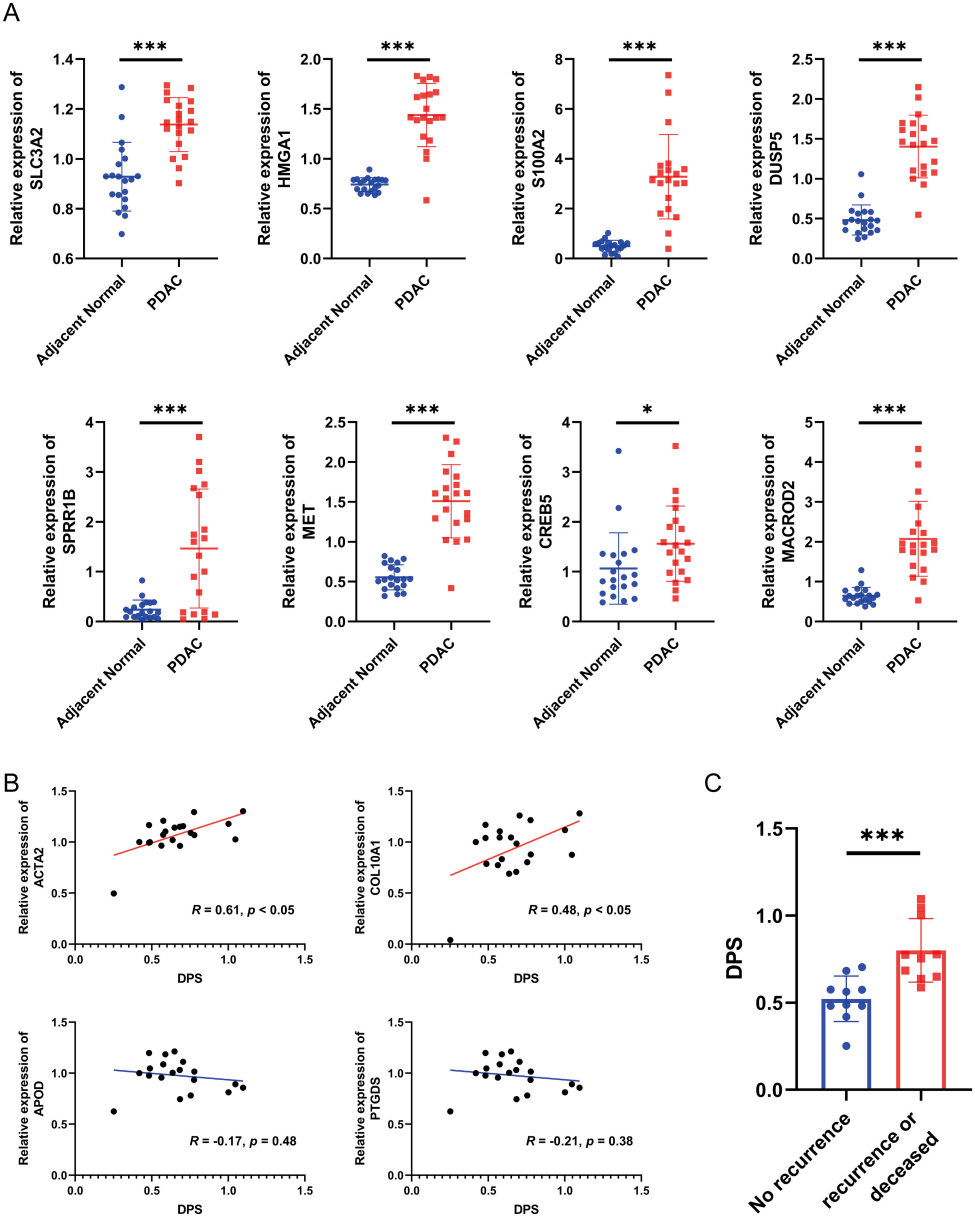
Validation of the disulfidptosis-related prognostic signature using biospecimens. **(A)** The relative expression levels of SLC3A2, HMGA1, S100A2, DUSP5, SPRR1B, MET, CREB5 and MACROD2 in 20 pairs of PDAC samples and adjacent normal tissues. **(B)** The correlation between the DPS and the expression of ACTA2, COL10A1, APOD and PTGDS. **(C)** The DPS of each PDAC patient in the “no recurrence” and “recurrence or deceased” groups. *P < 0.05, ***P < 0.001.

Since the establishment and validation of the disulfidptosis-related prognostic signature were heavily based on public databases, the generality of the signature is not very convincing. Hence, we collected a total of 20 pairs of PDAC samples and adjacent normal tissues from Shanghai East Hospital Biobank. The expression of eight genes in the signature, two myCAF markers (ACTA2 and COL10A1), and two iCAF markers (APOD and PTGDS) were determined by qRT-PCR. As a result, compared to the adjacent normal tissues, the expression levels of genes that constitute the disulfidptosis-related signature were significantly higher in PDAC samples (**Figure 8A**, all P < 0.05). Then, the DPS of each patient was calculated using the established formula above. We observed a significant positive correlation between the DPS and the expression levels of the two myCAF markers and no obvious relationship between DPS and the two iCAF markers (**Figure 8B**), agreeing with the hypothesis that myCAFs instead of iCAFs play a crucial role in fostering the development of pancreatic tumor cells. To evaluate the prognostic value of DPS in our cohort, we divided the patients into two groups. Patients who survived longer than 1 year with no recurrence after surgery were assigned to the “no recurrence” group, while patients who had tumor recurrence or deceased within one year were classified into the “recurrence or deceased” group. As shown in **Figure 8C**, the DPS of patients in the “recurrence or deceased” group was distinctly higher (P < 0.001), demonstrating the disulfidptosis-related prognostic signature worked well in PDAC patients’ risk stratification.

## 4 Discussion

Despite significant strides in the molecular understanding of PDAC in recent years, the prognosis remains exceedingly grim, primarily attributed to late-stage diagnosis and limited therapeutic options (40). Existing evidence revealed that *KRAS*, *CDKN2A*, *TP53*, and *SMAD4* gene mutations are the four major driver alterations for PDAC (41). Among them, *KRAS* is the only acknowledged oncogene while the others are generally characterized as tumor suppressor genes. The Cancer Genome Atlas Research Network has reported a staggering 93% mutation rate of *KRAS* in pancreatic cancer, underscoring its pivotal oncogenic role in PDAC tumorigenesis and progression (36). Although some certain KRAS inhibitors showed promising clinical efficacy (e.g. KRASG12C inhibitor, adagrasib, in non-small-cell lung cancer harboring a *KRAS^G12C^* mutation (42)), their effectiveness in PDAC necessitates further validation through clinical trials. Chemotherapy remains the cornerstone of PDAC treatment, and initiation immediately upon diagnosis is crucial to maximizing potential benefits. Liposomal irinotecan, exhibiting an enhanced permeability and retention effect in cancer and a longer half-life compared to nonliposomal irinotecan, has recently entered the market for advanced PDAC treatment (43). However, the prognosis of this disease is still very poor. Even worse, PDAC is considered one of the most immune-resistant tumor types, and a lot of single-agent immune modulators have been proven clinically ineffective (44). Thus, it is essential to discover novel molecular biomarkers to assist PDAC clinical management and unravel the underlying mechanisms.

Disulfidptosis is a newly discovered formation of programmed cell death, which enables cells to regulate their fates and coordinate their existence to benefit the living organism (6, 45). From our perspective, disulfidptosis can be succinctly described as a condition where limited NADPH production, resulting from glucose starvation’s inability to counteract the excessive uptake of cystine mediated by the overexpression of SLC7A11, leads to the accumulation of disulfide bonds, the collapse of the actin filament network, and ultimately, cell death. In recent years, numerous types of programmed cell death, including apoptosis, pyroptosis, ferroptosis, cuprotosis, and disulfidptosis, have been unveiled. However, translating these discoveries into clinical applications poses significant challenges. Therefore, we sought to explore potential applications in the clinical management of PDAC and elucidate the potential factors in this study. We noted that elevated expression of SLC7A11 is a pivotal factor in the initiation of disulfidptosis. To our surprise, the expression of SLC7A11 was almost solely high in a small subset of PDAC cells, convincing us of the occurrence of disulfidptosis in PDAC. Utilizing CRISPR-Cas9 screening results from a previous study (6), we identified a subset of tumor cells whose marker genes were correlated to unfolded protein and ferroptosis, providing corroborative evidence for the disulfidptosis features of the selected cells. In our interpretation, the unfolded protein may signify the collapsed actin filament network. Besides, the shared characteristics between ferroptosis and disulfidptosis involve insufficient reducing power and the imbalance of redox reactions (6, 7).

Navigating clinical decision-making poses significant challenges, particularly when determining the optimal regimen for a specific PDAC patient and identifying therapies that can enhance both longevity and quality of life. To address this pivotal question, our investigation focused on identifying prognostic markers for PDAC patients to facilitate effective risk stratification. Through integrated analysis on a total of 1,250 bulk RNA sequencing profiles and microarray data as well as extensive modeling efforts, we successfully obtained an 8-gene disulfidptosis-related prognostic signature and subsequently calculated the DPS of each PDAC patient. The DPS exhibited strong performance in both internal and external validation cohorts, highlighting its robustness and high efficiency. Moreover, we established a nomogram by integrating the DPS and clinical characteristics, which demonstrated top-notch AUC values and may offer valuable support for clinical decision-making. Honestly, with the development of bioinformatics and increasement of public sequencing data, many researchers have tried to construct prognostic models to predict the survival of patients and wish to improve the prognosis. However, these efforts often face various shortcomings. Guo et al. constructed a 3-gene ubiquitination-related signature associated with prognosis in PDAC. However, the study’s limited sample size warrants cautious interpretation (46). Chen et al. established a 7-gene hypoxia- and immune-related prognostic signature for PDAC, yet the underlying mechanisms were not investigated (47). Fang et al. built a 12-gene unfolded protein response-associated prognostic signature for PDAC, while its complexity hinders practical application in a clinical setting.

As for components of the disulfidptosis-related prognostic model we built, there were eight genes namely *SLC3A2*, *HMGA1*, *S100A2*, *DUSP5*, *SPRR1B*, *MET*, *CREB5*, and *MACROD2*. *SLC3A2* encodes a cell surface, a transmembrane protein belonging to the solute carrier family. It is reported that SLC3A2 forms a complex with SLC7A11 to constitute the system xc-cystine/glutamate antiporter (48), which was demonstrated indispensable in disulfidptosis (6). Several studies indicated that SLC3A2 mediates integrin signaling and drives integrin-dependent cancer cell behavior (49, 50), which is consistent with the crosstalk between myCAFs and tumor cells. HMGA1 is a chromatin-associated protein participating in various cellular processes including regulation of inducible gene transcription, DNA replication, and the metastasis of cancer cells. Recently, HMGA1 has been reported to induce FGF19 expression and drive PDAC tumorigenesis and stroma formation (51), which agrees with the enriched fractions of myCAFs in the TME of PDAC with high DPS. S100A2 was found to play an important role in cytoskeleton organization and epithelial-mesenchymal transition (52), reflecting the malignant hallmarks. DUSP5 belongs to the dual specificity protein phosphatase subfamily and inactivates ERK1/2, suppressing cell proliferation. Interestingly, it has been found that DUSP5 could suppress F-actin rearrangement (53), suggesting that DUSP5 is a mediator in disulfidptosis. SPRR1B is an envelope protein of keratinocytes and an acknowledged squamous differentiation marker (54). Our study showed that PDAC samples with high DPS were characterized by keratinization and filament organization, which may result from the high expression of SPRR1B. *MET* gene encodes a protein named hepatocyte growth factor receptor, which is a single-pass transmembrane tyrosine kinase receptor essential for embryonic development, organogenesis, and wound healing. It is reported that aberrantly active MET triggers tumor invasion, angiogenesis, and metastasis in several cancer types (55). Our results highlighted that PDAC samples with high DPS showed malignant hallmarks including cell proliferation and epithelial-mesenchymal transition, which may be due to the high MET expression. CREB5 is a member of the cAMP response element-binding protein family. Existing evidence revealed that CREB5 can directly activate MET, promoting cancer invasion and metastasis (56). MACROD2 is a deacetylase involved in removing ADP-ribose from mono-ADP-ribosylated proteins. Existing evidence demonstrated that loss of MACROD2 represses PARP1 activity and promotes chromosome instability and tumorigenesis (57, 58). However, other researchers came to the opposite conclusion. Morassa et al. found that MACROD2 overexpression mediated estrogen-independent growth and tamoxifen resistance in breast cancers (59). Hence, the role of MACROD2 is controversial in carcinogenesis. However, the expression of these 8 genes provided little information to dissect the underlying mechanisms of the DPS. Therefore, we turned our focus to the TME for a more comprehensive understanding.

The TME is typically composed of blood and lymphatic vascular networks, immune cells, stromal cells, extracellular matrix, and secreted molecules (60). In our study, both TIDE and CIBERSORT algorithms revealed significantly low levels of infiltrating CD8+ T cells in PDAC samples with high DPS. This observation indicated that PDAC patients with high DPS may lack the foundational elements for a response to ICB. ICB, a form of immunotherapy targeting molecules like CTLA-4, PD-1, PD-L1, and LAG-3, aims to enhance the immune system’s recognition and attack on cancer cells. ICB has shown success in the treatment of various solid tumors, particularly melanoma and non-small cell lung cancer (61). However, the application of ICB in PDAC is not as so satisfactory and still has a long way to go. The single-cell RNA sequencing profiles not only confirmed the very low fractions of CD8+ T cells in samples with high DPS but also provided another crucial insight: myCAFs accounted for the vast majority except for tumor cells. CAFs are the most prominent cellular component in the stroma of PDAC and secrete abundant extracellular proteins including collagens and fibronectin, supporting tumor development and contributing to drug resistance by acting as a “biological barrier” (62). Increasing evidence showed that CAFs, a cell type within the TME, stimulate angiogenesis and facilitate the proliferation and metastasis of cancer cells by remodeling the extracellular matrix and secreting cytokines (63, 64). Elyada et al. classified CAFs into three subtypes including myCAFs, iCAFs, and apCAFs (29). Among them, myCAFs, situated adjacent to cancer cells, are characterized by high αSMA (encoded by *ACTA2*) expression, activated TGFβ/SMAD2/3 signaling, and activation of transcription factors such as TWIST1, ZEB1, SNAI1, and SOX4, promoting a mesenchymal cell state. The iCAFs are discovered in the desmoplastic areas of cancer, farther away from the tumor cells, and had features including low expression levels of αSMA but upregulated cytokines and chemokines and activated IL1/JAK-STAT3 signaling pathway. The apCAFs were highlighted with expression of MHC class II-related genes and could induce T-cell receptor ligation. Consequently, we hypothesized that it is myCAFs that played an essential role in fostering tumorigenesis and development. Targeting CAFs, particularly myCAFs, may be a promising therapeutic strategy. However, the cautious approach is necessary, as merely ablating the stroma may inadvertently facilitate PDAC progression. Phase III trials evaluating the combination of PEGPH20, an enzyme degrading hyaluronic acid, a major component of the PDAC extracellular matrix (ECM), and nab-paclitaxel/gemcitabine faced setbacks (65). Thus, targeted strategies against CAFs should proceed judiciously.

However, our research also had some limitations. First, the causal relationship between disulfidptosis markers and immune evasion remained unproven, which weakened the rationale for targeting disulfidptosis in cancer treatment. Second, our validation cohort is not sufficiently large, which may lead to an overestimation of the model’s performance. In addition, the causal relationship between disulfidptosis and myCAFs needs further validated by mouse models and PDAC organoids.

In summary, we established an 8-gene disulfidptosis-related prognostic signature that demonstrated robust performance across various PDAC cohorts. In addition, the DPS, as calculated by the model, could provide insights into the PDAC TME and offer predictions on potential benefits from ICB treatment. For the easy calculation of the DPS and better clinical application, we created an online tool (https://mingshsmu.shinyapps.io/dps_pdac/). Furthermore, the abundance of myCAFs in the TME may be in connection with minimal immune cell infiltration and reduced responsiveness to ICB. Our study sheds light on the role of disulfidptosis in cancer clinical management and holds promise for enhancing the survival outcomes of patients with PDAC.

## Data Availability

The original contributions presented in the study are included in the article/**Supplementary Material**. Further inquiries can be directed to the corresponding authors.
