# Supplementary material for: Prognostic and immunotherapeutic potential of disulfidptosis-associated signature in pancreatic cancer
Source: Front Immunol. 2025 Mar 26;16:1568976. doi: 10.3389/fimmu.2025.1568976 (PMC11979277; doi:10.3389/fimmu.2025.1568976)
Supplement: Supplementary file 2 [file DataSheet1.docx]

**Supplementary Materials**

**Supplementary Figures**


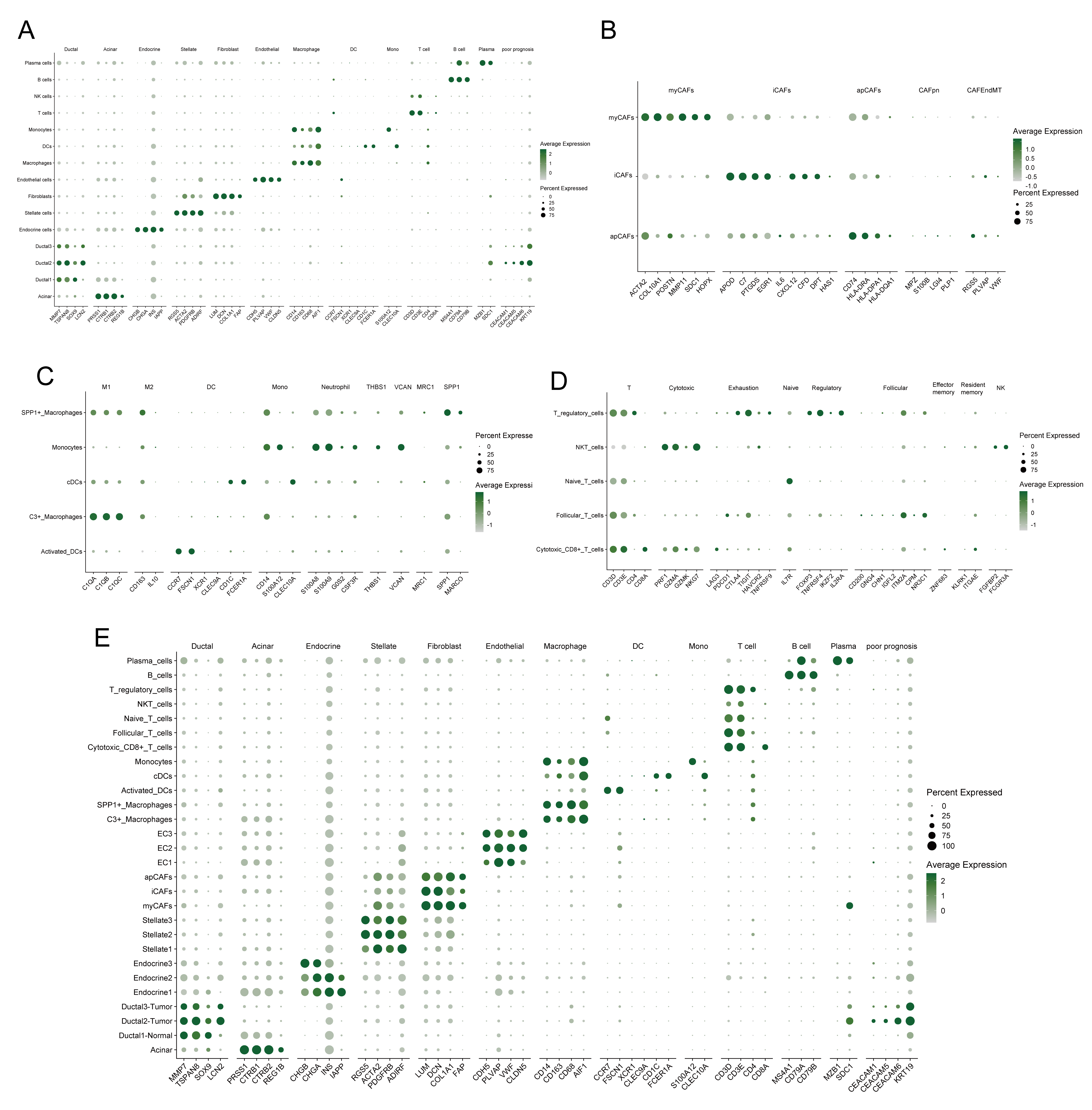


**Fig. S1** Markers across cell clusters. **(A)** The bubble plot shows cell type-specific markers across all cell clusters. **(B-D)** Bubble plots exhibit specific markers of CAFs **(B),** myeloid cells **(C)**, and T cells **(D)** across related cell clusters. **(E)** The bubble plot shows cell type-specific markers across all cell subpopulations.


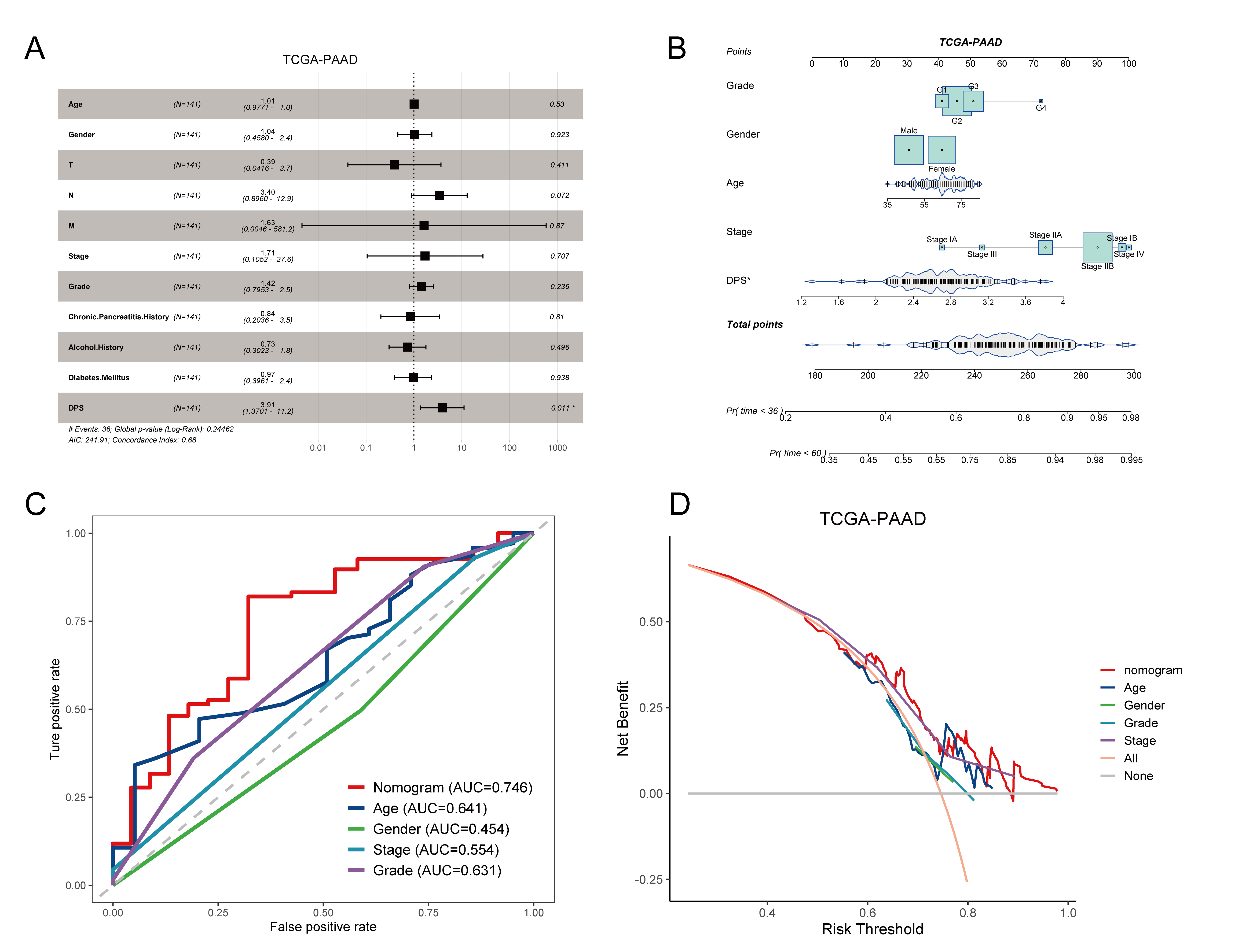


**Fig. S2** Evaluation of the prognostic significance of disulfidptosis-related score in TCGA-PAAD cohort. **(A)** The forest plot shows a multivariate Cox regression analysis of DPS and clinical characteristics in the TCGA-PAAD cohort. **(B)** A nomogram for patient risk stratification. **(C)** ROC curves demonstrate the predictive efficiency of nomogram, and clinicopathological features at 3-year-survival. **(D)** DCA curves show the net benefit of nomogram, and clinicopathological features for patients in TCGA-PAAD cohort at 2-year-survival and 3-year-survival. **P < 0.01; ***P < 0.001.


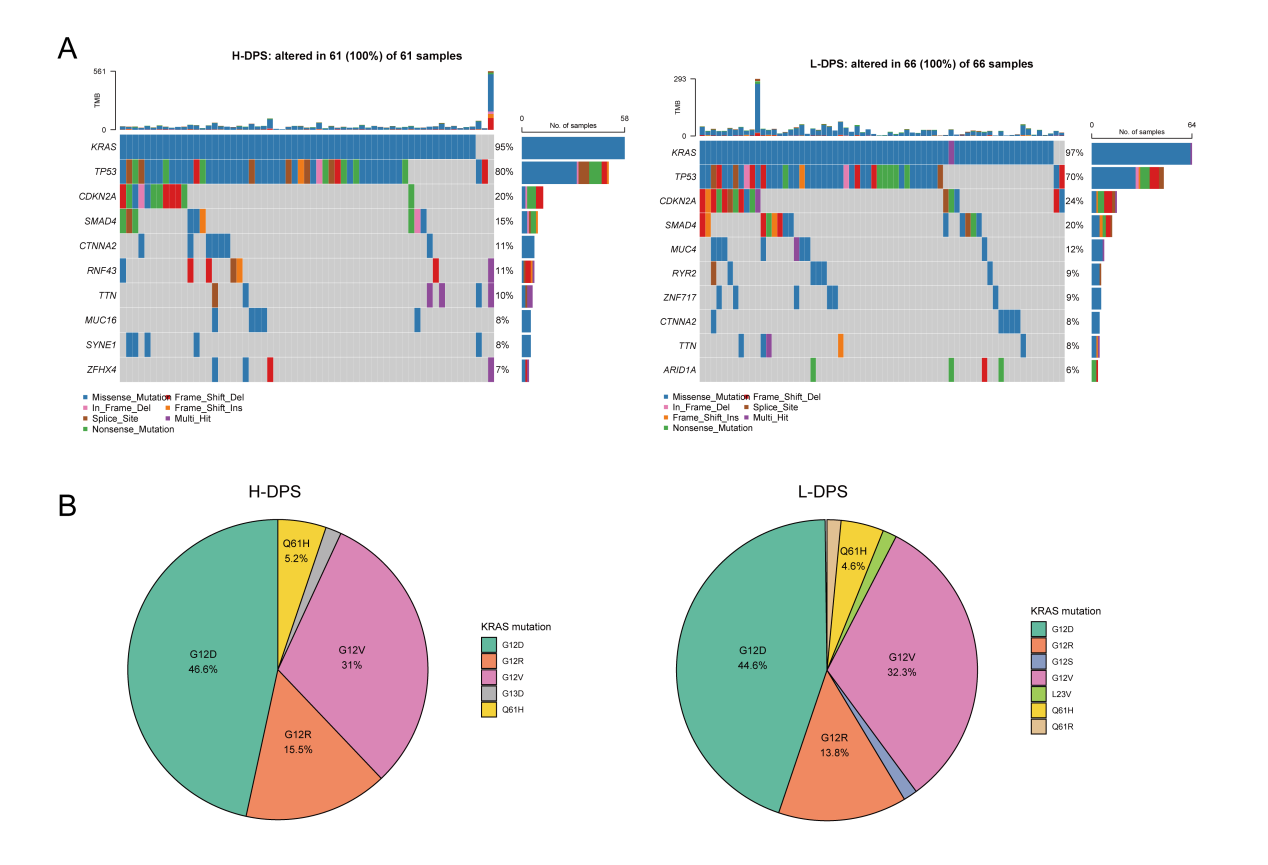


**Fig. S3** Exploration of the underlying mechanisms of DPS in gene mutation level. **(A)** Landscapes of top 10 frequently mutated genes in H-DPS and L-DPS groups. **(B)** Pie charts demonstrate the frequency of KRAS mutation types.


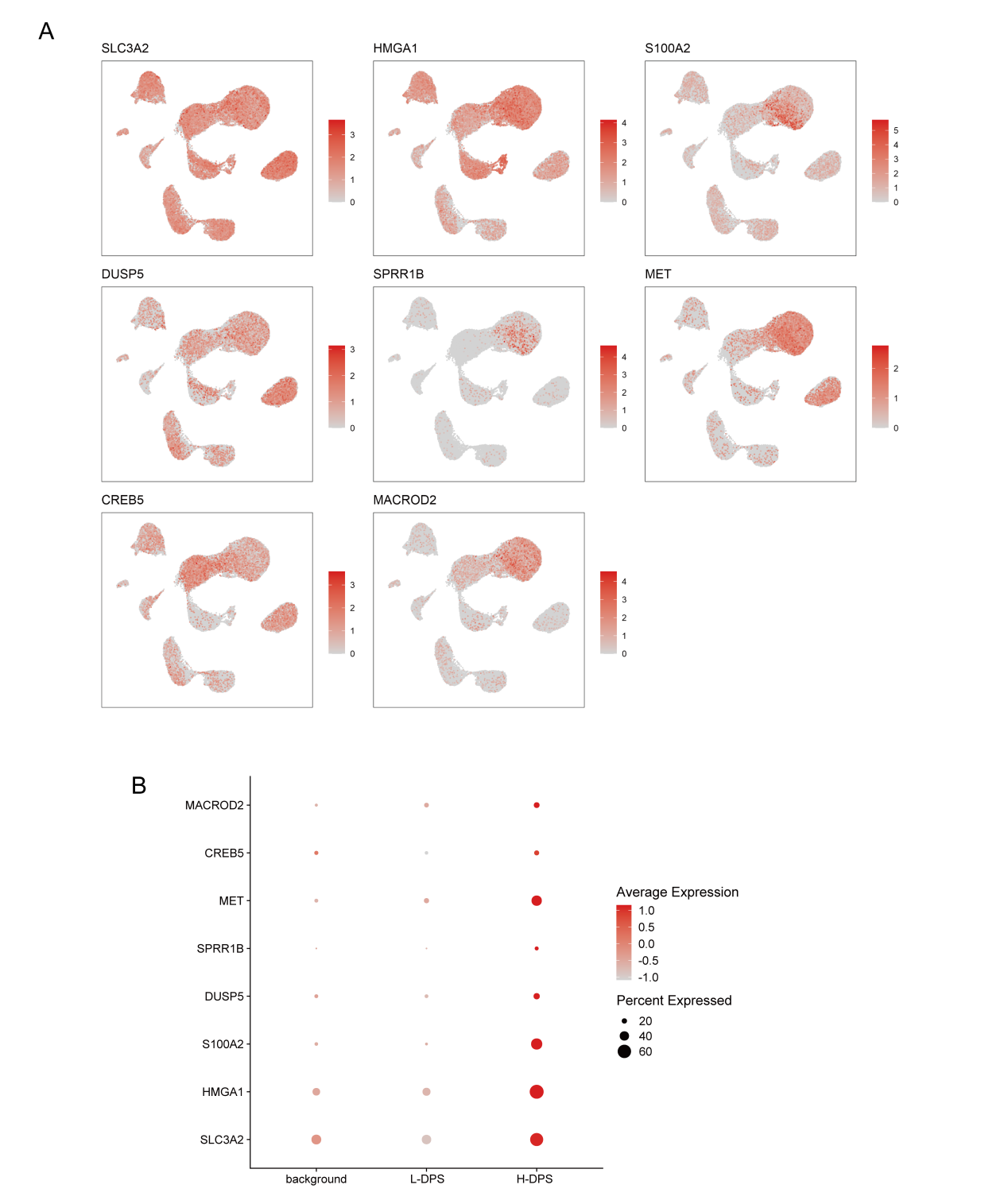


**Fig. S4** The expression of eight genes constituting the DPS-related prognostic model. **(A)** The expression levels and distribution of eight DPS-related genes are plotted on the UMAP map. **(B)** The bubble chart demonstrates the expression of eight DPS-related genes in H-DPS and L-DPS groups.


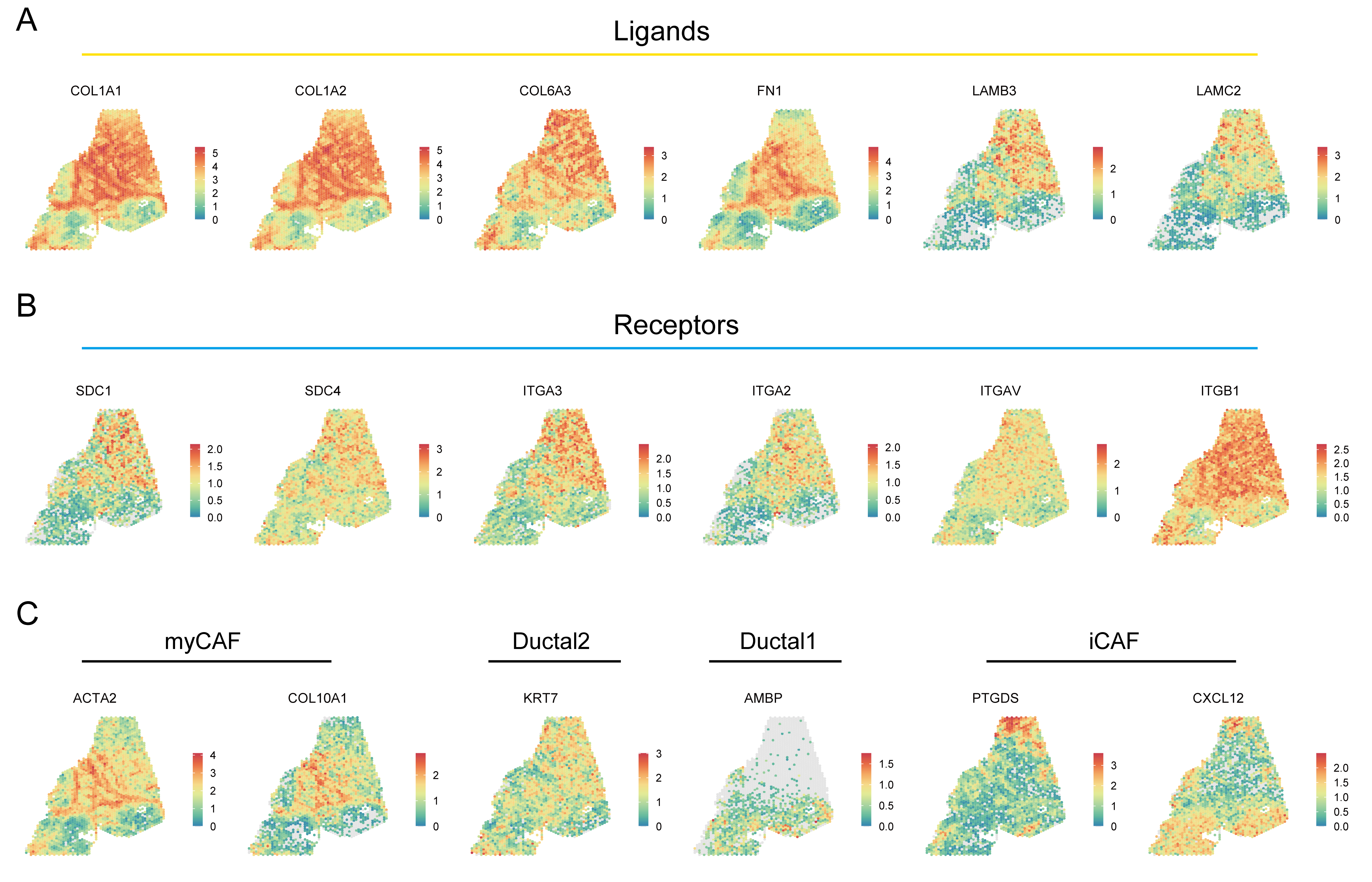


**Fig. S5** Spatial expression maps of representative ligands, receptors, and cell markers in PDAC sample A. **(A)** Spatial expression maps of representative ligands from collagen, laminin, and FN1 signaling pathways. **(B)** Spatial expression maps of representative receptors from collagen, laminin, and FN1 signaling pathways. **(C)** Spatial expression maps of representative markers of myCAFs, iCAFs, normal ductal cells (ductal1), and tumor cells (ductal2). iCAF: inflammatory cancer-associated fibroblast.


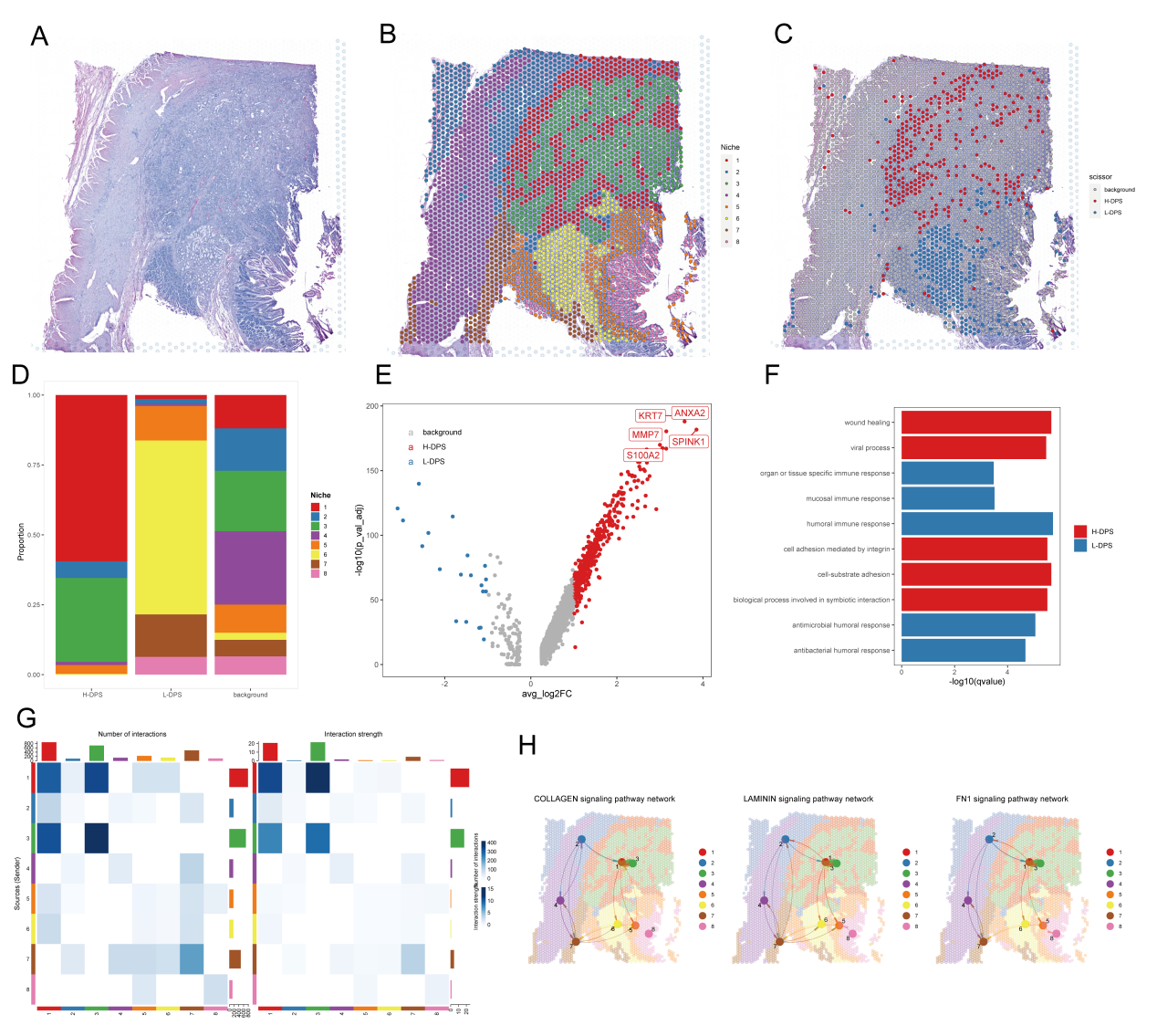


**Fig. S6** Investigation of the TME using spatial transcriptome data. **(A)** H&E staining for PDAC sample B. **(B)** Niches of PDAC sample B mapping with H&E staining. **(C)** H-DPS and L-DPS associated spots mapping with H&E staining. **(D)** Stacked bar plots exhibit the proportions of niches associated with H-DPS and L-DPS. **(E)** The volcano map shows DEGs between H-DPS and L-DPS-associated niches. The top five DEGs in H-DPS-associated niches were labeled with symbols. **(F)** Bar plots exhibit the top enriched terms in H-DPS and L-DPS-associated niches. **(G)** Heatmaps demonstrate the interaction number and strength among various niches. **(H)** The top three signaling pathway networks among niches in PDAC sample B.


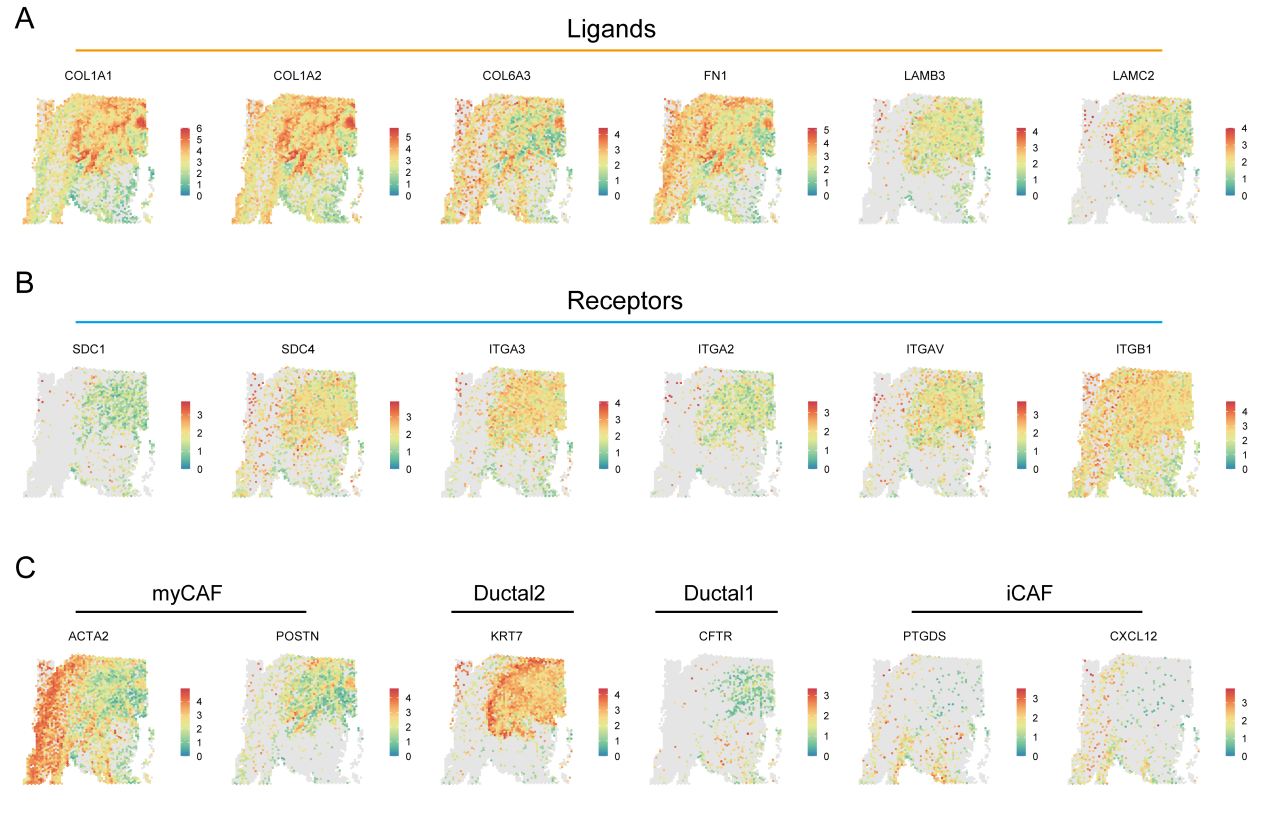


**Fig. S7** Spatial expression maps of representative ligands, receptors, and cell markers in PDAC sample B. **(A)** Spatial expression maps of representative ligands from collagen, laminin, and FN1 signaling pathways. **(B)** Spatial expression maps of representative receptors from collagen, laminin, and FN1 signaling pathways. **(C)** Spatial expression maps of representative markers of myCAFs, iCAFs, normal ductal cells (ductal1), and tumor cells (ductal2).


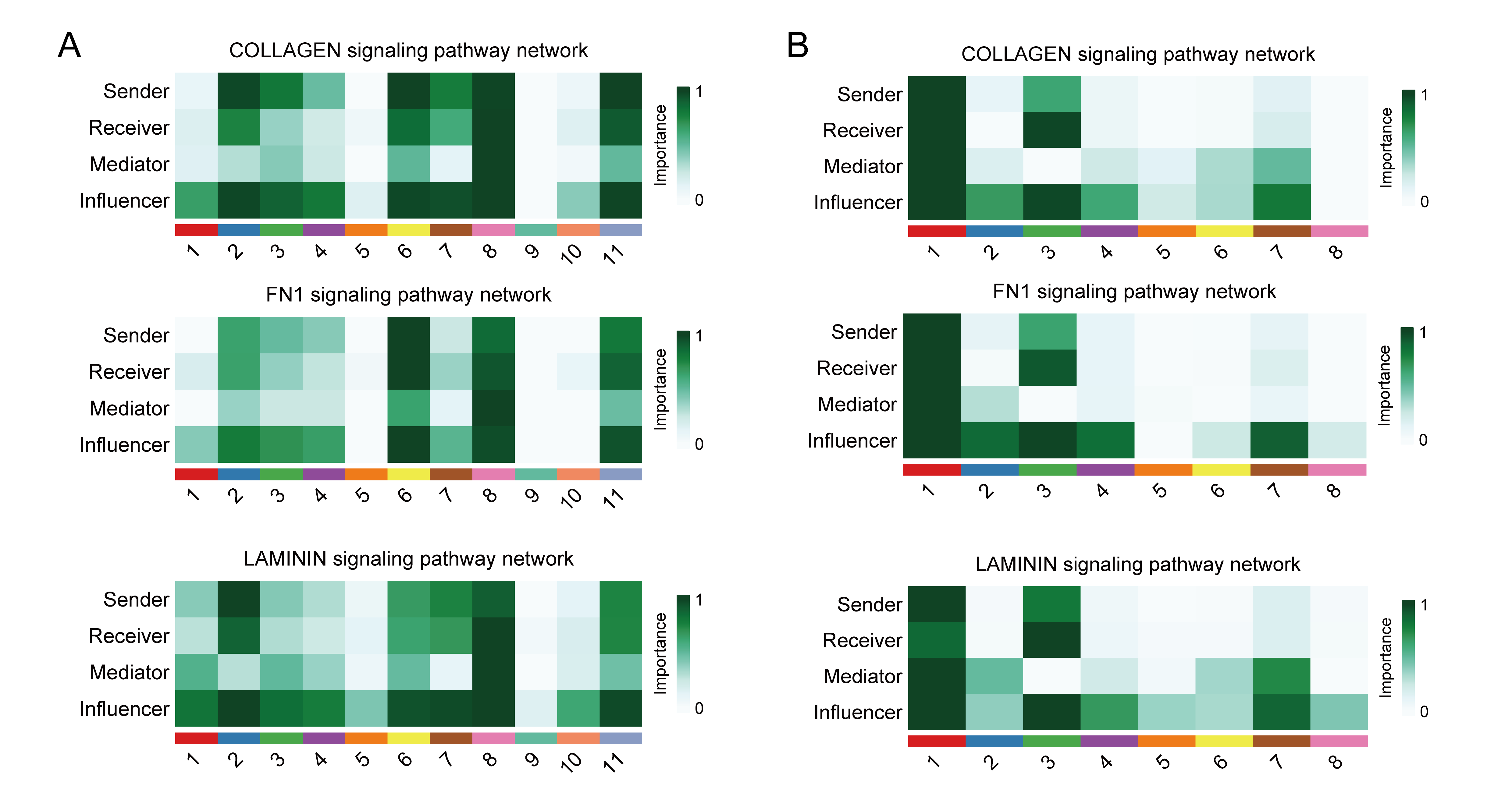


**Fig. S8** Heatmaps demonstrate the network centrality scores of the collagen, FN1, and laminin signaling pathways in PDAC sample A **(A)** and B **(B)**.

**Supplementary Tables**

**Table S1** Primers used in this study.

**Table S2** Significantly highly expressed genes in tumor cells with high disulfidptosis tendency compared with those with low disulfidptosis tendency. And these genes were called disulfidptosis-related genes (DPGs).

**Table S3** Enriched GO terms based on DPGs.

**Table S4** Enriched KEGG pathways based on DPGs.

**Table S5** Univariate Cox regression analysis of DPGs on the basis of integrated cohort.

**Table S6** The coefficients of eight selected genes constituting the disulfidptosis-related prognostic model.

**Table S7** Differentially expressed genes between PDAC samples in H-DPS and L-DPS groups.

**Table S8** TIDE scores of PDAC patients in the CPTAC-PDAC cohort estimated based on gene expression profiles.

**Table S9** Fractions of 22 immune cell types calculated by the CIBERSORT algorithm based on gene expression profiles from the CPTAC-PDAC cohort.

**Table S10** Detailed interactions between myCAFs and PDAC tumor cells.

**Table S11** Markers of H-DPS and L-DPS associated niches in PDAC sample A.

**Table S12** Markers of H-DPS and L-DPS associated niches in PDAC sample B.
